# Nanoscale engineering of gold nanostars for enhanced photoacoustic imaging

**DOI:** 10.1186/s12951-024-02379-7

**Published:** 2024-03-16

**Authors:** Rui Zhang, Sven Thoröe-Boveleth, Dmitry N. Chigrin, Fabian Kiessling, Twan Lammers, Roger M. Pallares

**Affiliations:** 1 Institute for Experimental Molecular Imaging, RWTH Aachen University Hospital, 52074 Aachen, Germany; 2 Institute for Occupational, Social and Environmental Medicine, Medical Faculty, RWTH Aachen University, 52074 Aachen, Germany; 3 Institute of Physics (1A), RWTH Aachen University, 52056 Aachen, Germany; 4 DWI – Leibniz Institute for Interactive Materials, 52076 Aachen, Germany

## Abstract

Photoacoustic (PA) imaging is a diagnostic modality that combines the high contrast resolution of optical imaging with the high tissue penetration of ultrasound. While certain endogenous chromophores can be visualized via PA imaging, many diagnostic assessments require the administration of external probes. Anisotropic gold nanoparticles are particularly valued as contrast agents, since they produce strong PA signals and do not photobleach. However, the synthesis of anisotropic nanoparticles typically requires cytotoxic reagents, which can hinder their biological application. In this work, we developed new PA probes based on nanostar cores and polymeric shells. These AuNS were obtained through one-pot synthesis with biocompatible Good’s buffers, and were subsequently functionalized with polyethylene glycol, chitosan or melanin, three coatings widely used in (pre)clinical research. Notably, the structural features of the nanostar cores strongly affected the PA signal. For instance, despite displaying similar sizes (i.e. 45 nm), AuNS obtained with MOPS buffer generated between 2 and 3-fold greater signal intensities in the region between 700 and 800 nm than nanostars obtained with HEPES and EPPS buffers, and up to 25-fold stronger signals than spherical gold nanoparticles. A point source analytical model demonstrated that AuNS synthesized with MOPS displayed greater absorption coefficients than the other particles, corroborating the stronger PA responses. Furthermore, the AuNS shell not only improved the biocompatibility of the nanoconstructs but also affected their performance, with melanin coating enhancing the signal more than 4-fold, due to its own PA capacity, as demonstrated by both in vitro and ex vivo imaging. Taken together, these results highlight the strengths of gold nanoconstructs as PA probes and offer insights into the design rules for the nanoengineering of new nanodiagnostic agents.

**Supplementary Information:**

The online version contains supplementary material available at 10.1186/s12951-024-02379-7.

## Introduction

Photoacoustic (PA) imaging, also known as optoacoustic imaging, is a biomedical imaging modality where acoustic signal is generated by irradiating a target with light [1, 2]. It is a non-invasive imaging tool that is commonly used in preclinical research [3, 4], and it has been explored for clinical purposes [5–7]. Compared to fluorescent signal, the emitted acoustic waves are less scattered by tissue, providing higher resolution [8, 9]. Moreover, because certain clinically relevant endogenous targets display characteristic PA signals, such as oxygenated and deoxygenated hemoglobin, those can be selectively imaged and differentiated without the administration of a probe [10, 11]. Still, most PA imaging applications require a probe to generate the signal [6, 12]. To date, most probes are based on organic dyes, which suffer from photobleaching and limited absorbance and PA signal generation [10, 13]. Consequently, new probes with enhanced PA capabilities and resistance to photobleaching are necessary to further enhance the diagnostic capacities of PA imaging.

Gold nanoparticles are currently being explored as highly performing PA probes because of their strong extinction coefficients and morphology-dependent optical properties [3]. By modifying size and shape of the nanoparticles, their localized surface plasmon (LSP) resonances can be tuned [14–16]. Notably, upon light excitation, the nanoparticles partially release the absorbed energy through non-radiative decay pathways, in which large amounts of heat are locally generated [17, 18]. This release of heat has been exploited for diagnostics via PA imaging and therapeutics via photothermal therapy [19, 20]. For instance, gold nanoparticle-based photothermal therapy is currently being investigated in clinical studies for prostate cancer treatment and atherosclerotic plaque removal [21].

Among the different morphologies synthetically available, those with anisotropic features, such as rods and stars, are of particular interest, since their LSP bands can be centered within the near-infrared window (NIR, 650–1100 nm), i.e. a region of the spectrum where tissue displays lower absorption and scattering [22–26]. Gold nanorods have been historically preferred for photoacoustic imaging, since they were the first anisotropic nanoparticles obtained [27–29]. However, gold nanorods are primarily synthesized with cetyltrimethylammonium bromide as shape-directing agent [30], which is cytotoxic and hard to remove from the nanoparticle surface, potentially hampering the use of gold nanorods for biomedical applications [31, 32]. Alternatively, gold nanostars (AuNS) with LSP resonances that depend on nanoparticle morphology [33], such as aspect ratio of the branches, can been obtained with biocompatible reagents [34–36]. For example, the seedless synthesis of AuNS with Good’s buffers is a commonly used synthetic method, since it relies on biocompatible Good’s buffers, such as 4-(2-hydroxyethyl)piperazine-1-ethanesulfonic acid (HEPES), 4-(2-hydroxyethyl)-1-piperazinepropanesulfonic acid (EPPS), and 3-(N-morpholino)propanesulfonic acid (MOPS), which are commonly used in cell and tissue cultures. The Good’s buffers act as both reducing and shape-directing agents, tailoring the morphology of the AuNS cores and branches, and stabilizing the particles in aqueous solution without the need for further addition of surfactants [16, 35, 36]. The key features of AuNS, such as LSP resonance position, size and the morphology of branches can be easily modulated by the selection of Good’s buffer and the ratio of buffer to gold during the synthesis [16, 36]. Despite all these advantages, AuNS synthesized with Good’s buffers have been rarely (if ever) used for PA imaging.

Therapeutic and imageable gold nanoconstructs are regularly functionalized with polymers to improve their biocompatibility and stability [37, 38]. The polymer shell not only prevents gold nanostructures from aggregating in biological fluids but also enhances their circulation times in blood, improving their passive accumulation in pathological tissues via the enhanced permeability and retention effect [39, 40]. Notably, pioneering work by Repenko et al. demonstrated that the polymer coating could enhance the PA signal generated by gold nanoconstructs, as it changes the dielectric environment of the particles and some polymers display inherent PA response [41]. Thus, the polymer coating may offer a new avenue to further strengthen the PA intensity of AuNS synthesized with Good’s buffers. However, to that end, a systemic study of the effect of gold nanoparticle-ligand interactions on PA imaging performance is necessary.

Here, we show that the structural features of the gold core and the ligand shell play instrumental roles in the PA response of the probes. Although all synthesized nanoconstructs had similar sizes, the AuNS obtained with MOPS displayed between 2 and 3-fold greater PA signal than the stars obtained with HEPES and EPPS, and between 19 and 25-fold greater PA signal than the control spherical gold nanoparticles (AuNPs), which were chosen because they do not display anisotropic features and are the most commonly used gold nanocrystals for biomedical applications. Furthermore, while most ligands did not affect the imaging performance of the probes and only improved their biocompatibility, melanin enhanced the imaging response of the nanoconstructs up to 4.5-fold, because of its inherent optical characteristics. The PA generation of the melanin functionalized AuNS was preserved in biological environment as demonstrated by in vitro and ex vivo experiments. Our results provide novel insights into the design of highly performing PA probes for (pre)clinical research.

## Results and discussion

**Fig. 1:**
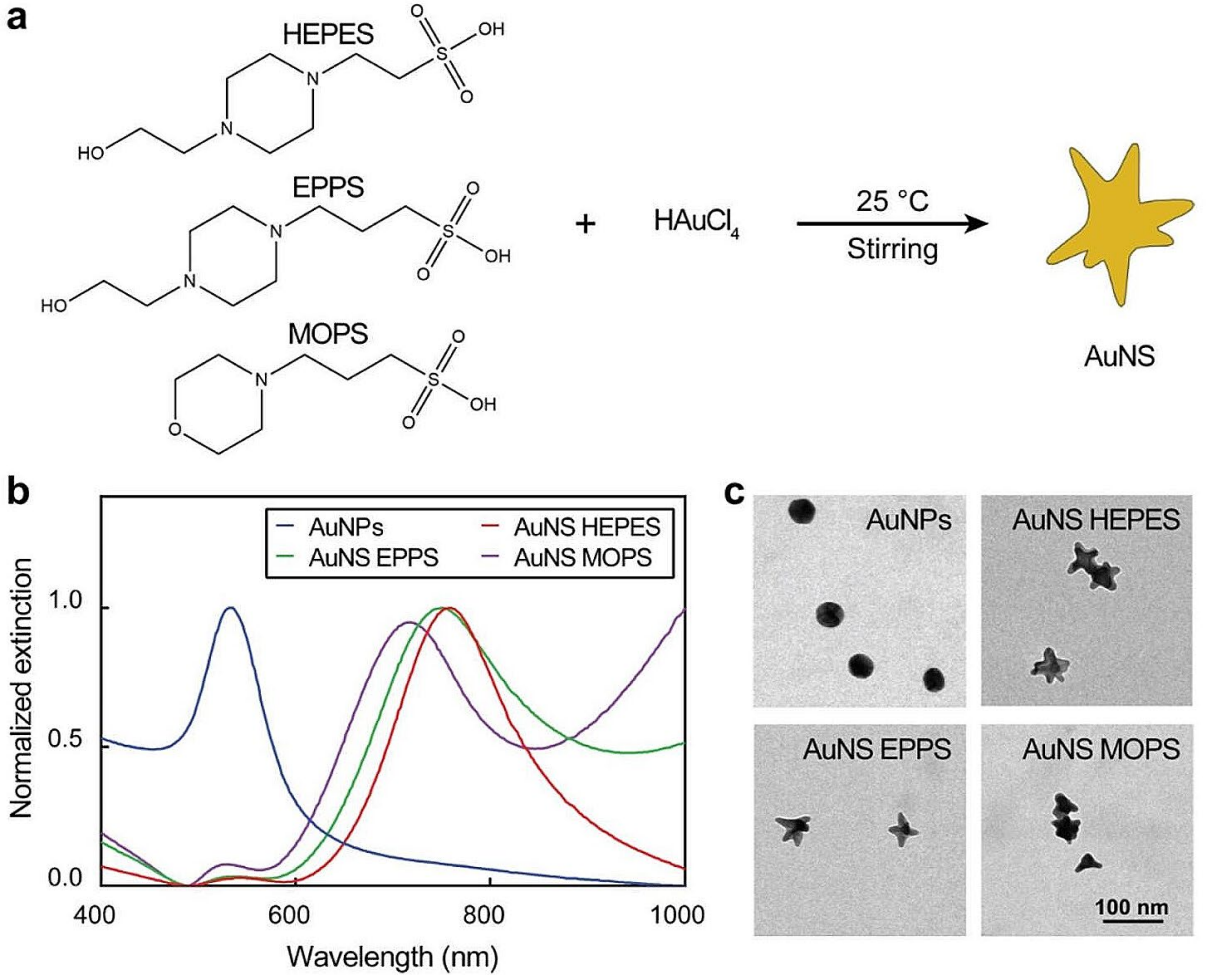
Seedless synthesis of AuNS with Good’s buffers. (**a**) Schematic representation of seedless growth of AuNS. (**b**) Extinction spectra and (**c**) TEM micrographs of AuNPs and AuNS

**Fig. 2:**
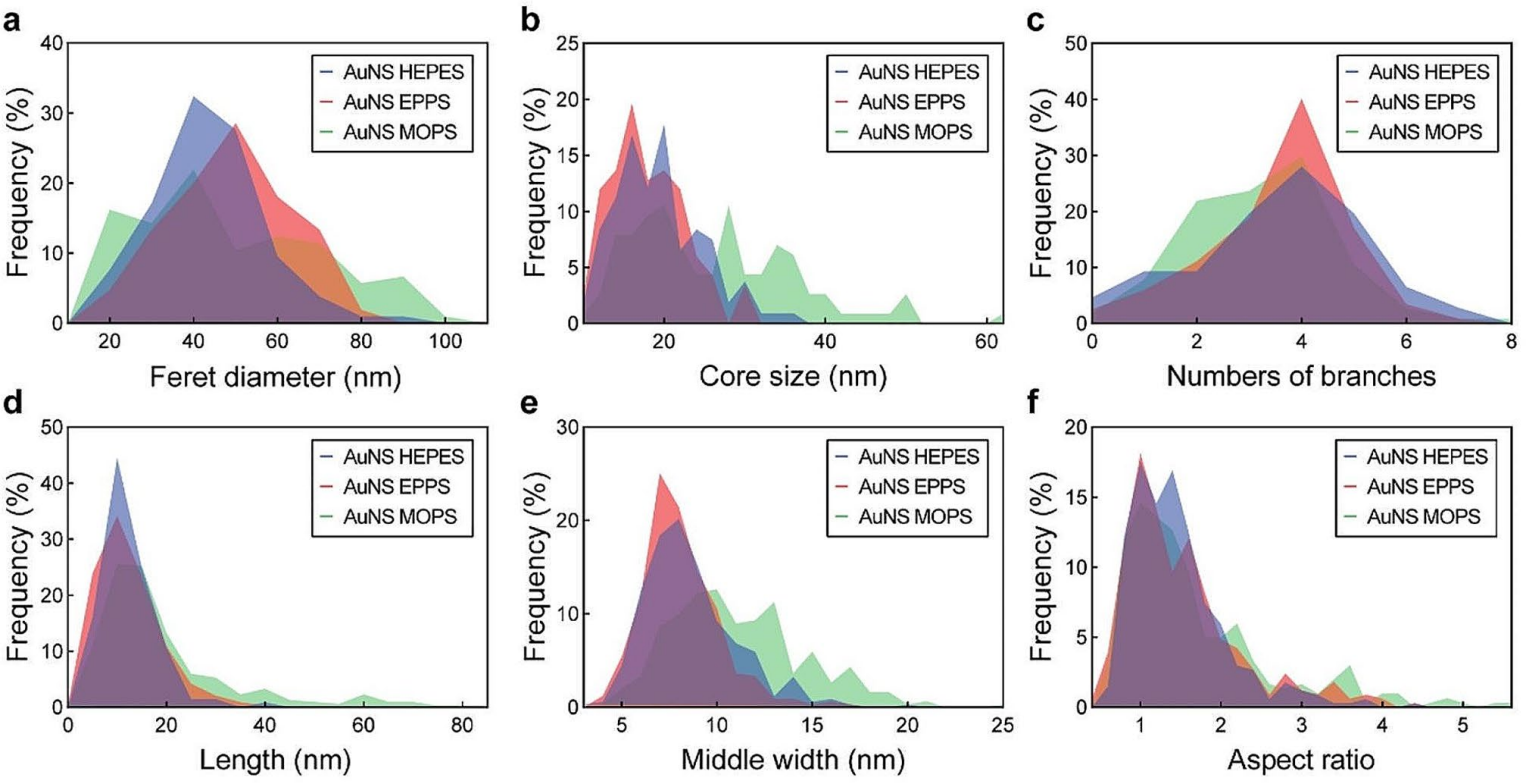
Geometrical analysis of AuNS synthesized with HEPES, EPPS or MOPS. Distributions of (**a**) Feret diameter, (**b**) core size, (**c**) numbers of branches, (**d**) branch length, (**e**) branch middle width and (**f**) branch aspect ratio of AuNS

AuNS were synthesized via one-pot synthesis with three different Good’s buffers, namely HEPES, EPPS, and MOPS (Fig. 1). To better compare the three different types of AuNS, the chemical conditions were tuned to produce AuNS with similar sizes (Feret diameters around 45 nm). The morphologies and branch dimensions of the AuNS were characterized by transmission electron microscopy (TEM). AuNS HEPES and AuNS EPPS showed similar morphological features, including core size, branch length and branch aspect ratio (Fig. 2 and S1, and Tables S1 to S4). Meanwhile, AuNS MOPS displayed larger cores and less homogeneous morphologies, as demonstrated by their wider distributions in size and branch characteristics. This observation was consistent with previous findings, which identified that AuNS obtained with MOPS were more heterogeneous compared to the particles obtained with HEPES or EPPS [36]. The AuNS obtained with HEPES and EPPS displayed LSP bands centered at 750 nm, while the LSP band of AuNS MOPS was centered at 720 nm. As a control, 45-nm spherical AuNPs were synthesized via seed-mediated growth with citrate.

For the functionalization of the nanoparticles, four stabilization ligands commonly used in nanomedicine, namely 2 and 6 kDa thiol-terminated polyethylene glycol (PEG), chitosan and melanin, were chosen. The thiol functional group of thiol-terminated PEG strongly binds to gold surfaces, while the polymeric part of the ligand provides high stability and biocompatibility to the nanoconstructs. As a result, gold nanoconstructs used in clinical settings are commonly functionalized with thiol-terminated PEG [21]. As a naturally existing polymer, chitosan has also been widely explored for drug delivery [42, 43]. The cationic polymer can encapsulate negatively charged gold nanostructures, providing a hydrophilic, biocompatible and functional layer [44, 45]. Regarding melanin, it can be obtained by the auto-oxidation of dopamine, forming a polymeric layer on the surface of gold nanostructures. Melanin displays a broad absorption that can be exploited in photothermal applications, such as PA imaging and photothermal therapy [46, 47]. Hence, the biomimetic melanin coating not only may improve the colloidal stability of the nanoconstructs, but it has the potential to enhance their PA signal.

**Fig. 3:**
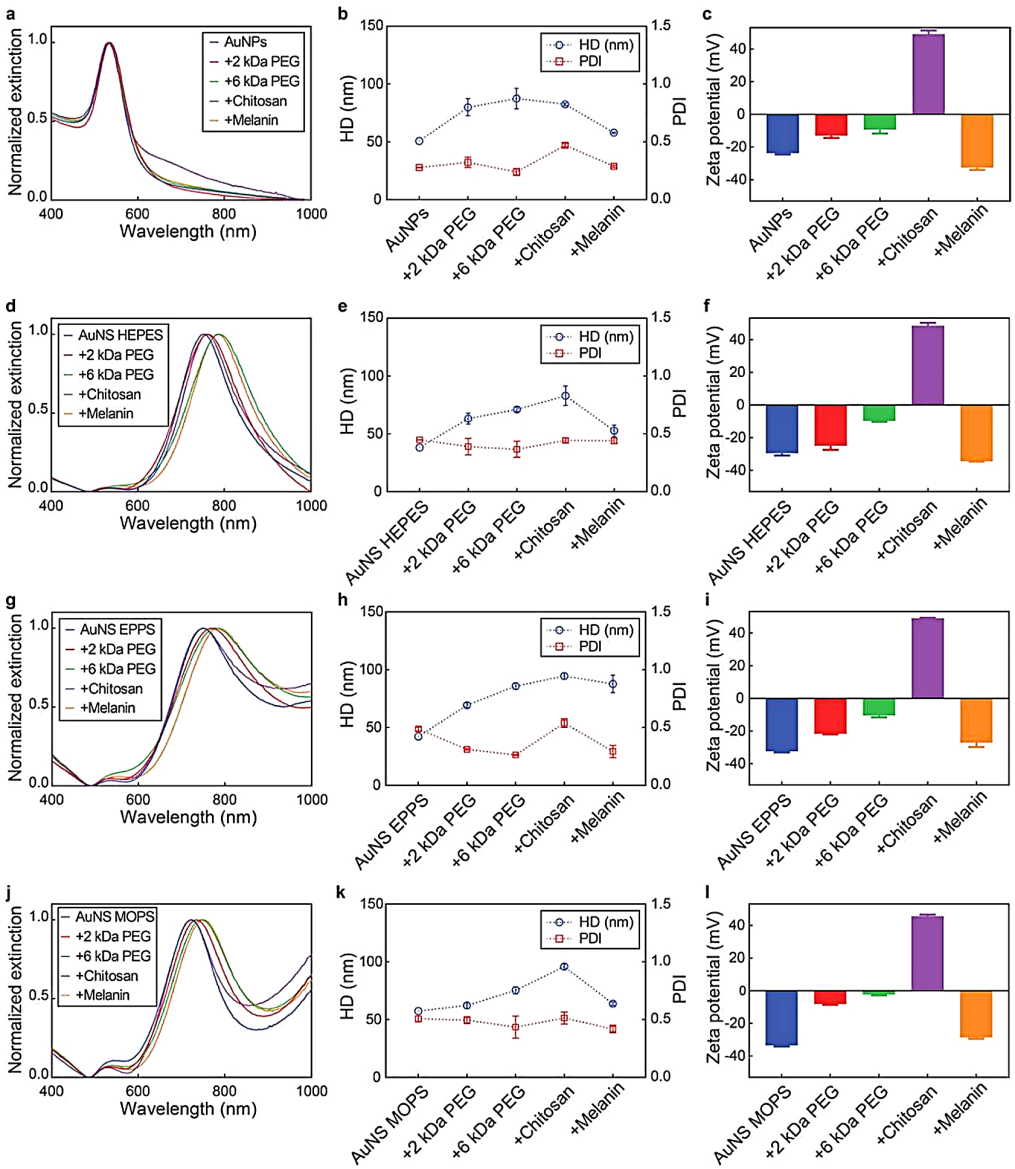
AuNP and AuNS characteristics before and after functionalization. (**a**, **d**, **g**, **j**) Extinction spectra, (**b**, **e**, **h**, **k**) hydrodynamic diameter (HD) and polydispersity index (PDI), and (**c**, **f**, **i**, **l**) zeta potential of AuNPs and AuNS before and after functionalization with 2 kDa PEG, 6 kDa PEG, chitosan and melanin. Values represent mean ± standard deviation of three different batches of nanoconstructs

Next, we optimized the functionalization of the gold nanoparticles with the different ligands. To that end, spherical AuNPs and AuNS synthesized with HEPES (as representatives of the star-shaped nanoparticles) were conjugated with the four ligands under different molar ratios (Figures S2 and S3). The functionalization of AuNPs with the different ligands slightly red-shifted their LSP band (from 534 to 536 nm), because of the change in the dielectric constant of the nanoparticle surroundings, and increased their hydrodynamic diameter (Fig. 3 and Figure S2). As expected, the 6 kDa PEG caused the largest increase in hydrodynamic size, as it was the largest polymer tested. Regarding zeta potential, the PEGylation coating shielded the negatively charged surface of the spherical AuNPs, causing charge neutralization (Figure S2). Meanwhile, surface functionalization with chitosan yielded positively charged nanoparticles due to the strong positive charge of the polymer. In all cases, the AuNP core was not altered after functionalization, as observed in the TEM micrographs (Figure S4). Interestingly, although carbon-based polymers display low contrast in TEM compared to gold (and other heavy elements), the melanin coating around the AuNP surface was visible in the micrographs. Similar optimization experiments were carried out for the functionalization of AuNS. In all cases, the LSP band was red-shifted and the hydrodynamic diameter of the particles increased after functionalization (Figure S3). Consistent with the AuNP results, 6 kDa PEG caused the greatest increases in hydrodynamic diameter. The melanin coating caused significant variations in the extinction spectra of AuNS HEPES and red-shifted the LSP band from 752 to 788 nm. The changes in zeta potentials after the functionalization of AuNS HEPES were consistent with the ones identified for AuNPs. Furthermore, no morphological changes were observed in the AuNS cores after functionalization (Figure S5). Considering the LSP band shifts, hydrodynamic size increases and zeta potential changes, the optimal functionalization conditions for AuNPs and AuNS HEPES were selected, as displayed in Table S5 and S6.

The functionalization conditions identified for AuNS HEPES were further applied to the nanostars obtained with EPPS and MOPS, yielding similar changes in hydrodynamic diameter, zeta potential and LSP bands (Fig. 3). Moreover, the successful functionalization of the nanoconstructs was further corroborated by FTIR spectroscopy (Figure S6). For example, the conjugation with PEG was confirmed by the presence of two characteristic bands between 2850 and 3000 cm^− 1^ (stretching vibration of -CH_2_) and at 1100 cm^− 1^ (stretching vibration of C-O-C) [48, 49]. The chitosan capping was evidenced by the presence of multiple characteristic bands, including a band observed between 3200 and 3500 cm^− 1^ (symmetric vibration of amine N-H and stretching vibration of O-H), another one located at 2895 cm^− 1^ (stretching vibration of C-H), and three bands positioned at 1656 cm^− 1^, 1591 cm^− 1^, and 1373 cm^− 1^ (amide groups) [50, 51]. The nanoparticle functionalization with melanin was verified by the existence of characteristic bands at 3410 cm^− 1^ (stretching vibration of phenolic O-H and N-H), at 1605 cm^− 1^ (stretching vibration of aromatic C-C and bending vibration of N-H), at 1510 cm^− 1^ (shearing vibration of N-H), and at 1295 cm^− 1^ (stretching vibration of phenolic C-O) [52, 53].

**Fig. 4:**
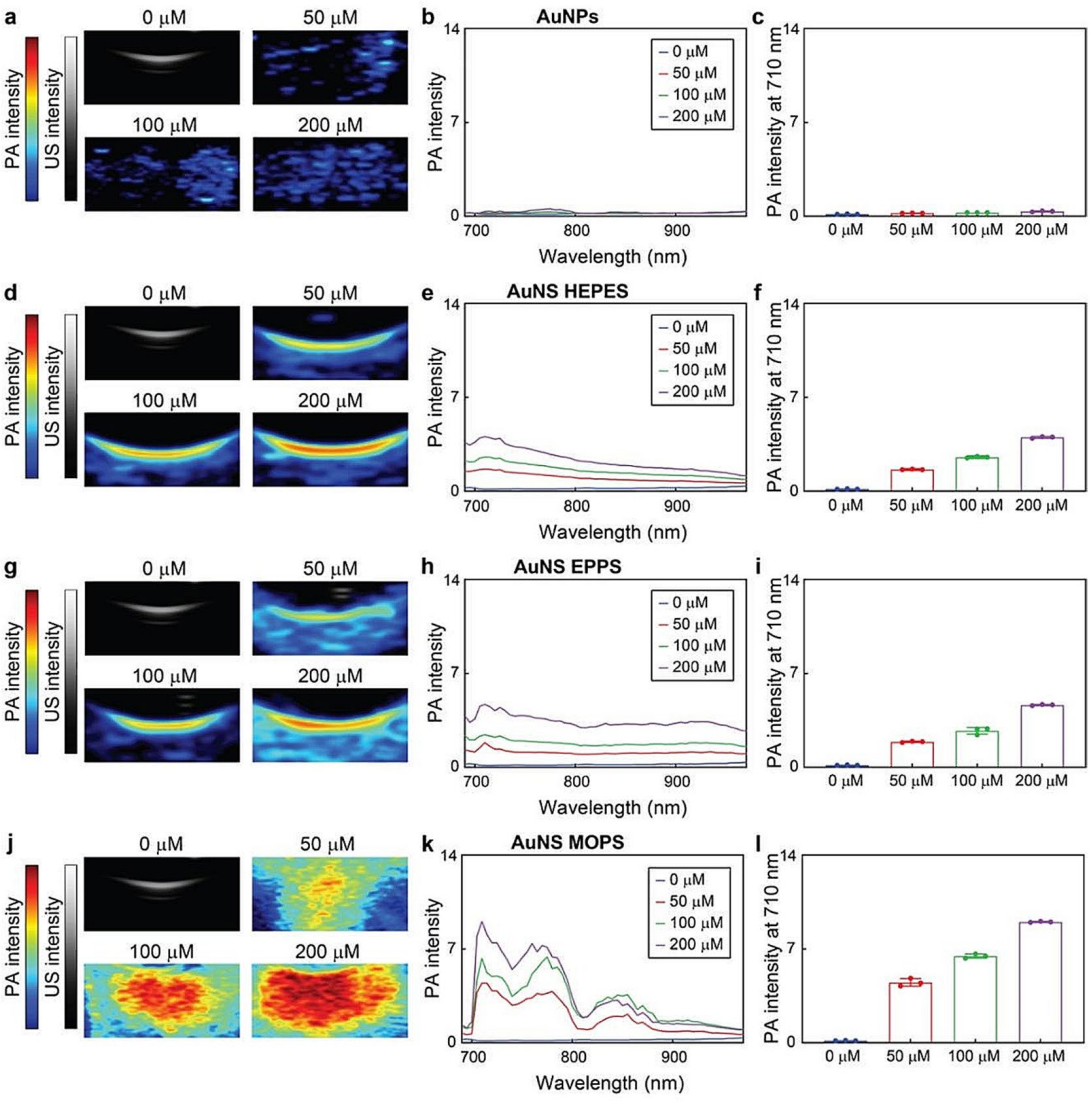
PA-US imaging of AuNPs and AuNS in gelatin phantoms. PA images, PA-US spectra and PA intensities at 710 nm of (**a**, **b**, **c**) AuNPs, (**d**, **e**, **f**) AuNS HEPES, (**g**, **h**, **i**) AuNS EPPS, and (**j**, **k**, **l**) AuNS MOPS at different gold concentrations (from 0 to 200 µM). Values in columns represent mean ± standard deviation of three different batches of nanoconstructs

Next, we evaluated the PA imaging characteristics of the four (uncoated) gold nanoparticles. Different concentrations of AuNPs and AuNS (HEPES, EPPS and MOPS) ranging from 0 to 200 µM were firstly imaged with a preclinical system that provides both bright-mode ultrasound (US) and PA, from 680 to 970 nm, responses in gelatin phantoms (Fig. 4, Figure S7a, and Figure S8). The AuNPs displayed weak PA responses, as a consequence of their low extinction in the NIR region of the spectrum. Both AuNS HEPES and EPPS exhibited considerable PA responses, which increased as a function of nanoparticle concentration (up to 4.0 ± 0.1 and 4.7 ± 0.1 at 200 µM gold, respectively). Notably, AuNS grown with MOPS buffer yielded the strongest PA response (9.0 ± 0.0 at 200 µM gold) among the tested nanoconstructs. Although all nanoparticles had similar average Feret diameters, AuNS MOPS were more heterogeneous and displayed wider size and branch distributions, presenting a greater proportion of nanoparticles with larger core sizes and longer branches (Tables S1 to S4). Larger particles are known to display greater overall absorption compared to their smaller counterparts [54], which may have contributed to the stronger PA responses of AuNS MOPS.

**Fig. 5:**
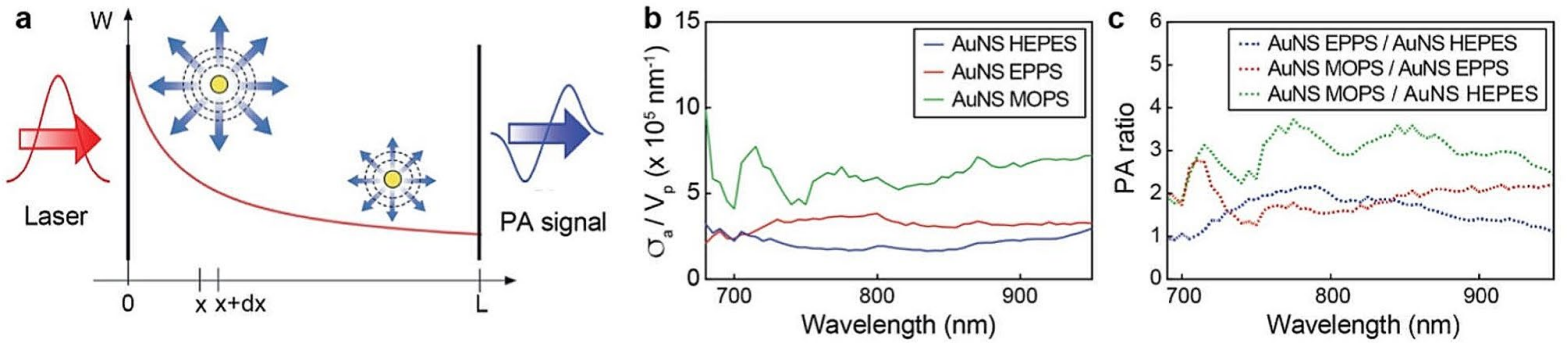
Modeling of light absorption efficiency and PA responses of AuNS. (**a**) Representation of PA intensity phenomenon as considered by the analytical model. Plasmonic nanoparticles generate heat by absorbing the photons of the laser radiation, generating ultrasound signal. The laser beam intensity (W) decreases as it propagates through the length (L) of the sample. Adapted with permission of ref 55. Copyright American Chemical Society 2020. (**b**) Absorption cross-section (σ_a_) per the nanoparticle volume (V_p_) of AuNS HEPES, EPPS and MOPS. (**c**) PA intensity ratio between AuNS HEPES, EPPS and MOPS

To further understand the different PA responses of the AuNS, we applied a photoacoustic point source model previously developed by us, that describes the PA intensity generated by a single plasmonic particle [41, 55]. The PA response depends on the nanoparticle absorption (σ_a_), extinction cross-sections and the laser intensity at the nanoparticle location (Fig. 5a). At the concentration regime in which our experiments were carried out, the total PA intensity is linearly dependent on concentration [55]. The model enables to reconstruct the wavelength dependence of σ_a_ per nanoparticle volume (V_p_) (Fig. 5b). AuNS MOPS displayed the highest absorption cross-section per nanoparticle volume in the tested wavelength range and, as a result, the PA signal generated by AuNS MOPS was higher than those of AuNS HEPES and EPPS (Fig. 5c). These predictions by the analytical model agreed with our experimental results, and identified the higher absorption cross-section per nanoparticle volume of AuNS MOPS as responsible for the stronger PA signals generated by the particles.

**Fig. 6:**
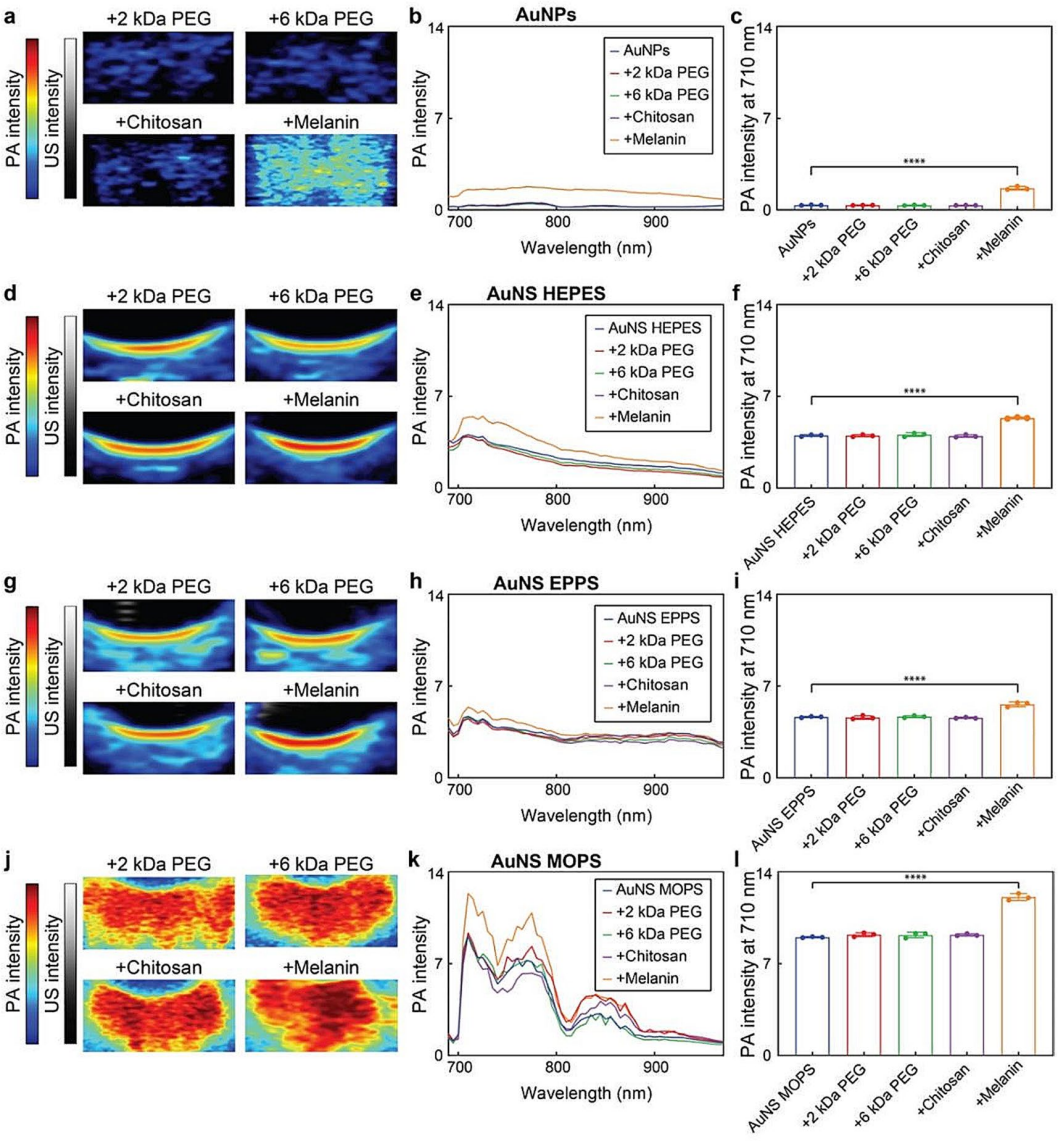
PA-US imaging of functionalized AuNPs and AuNS in gelatin phantoms. PA-US images, PA spectra and PA intensities at 710 nm of functionalized (**a**, **b**, **c**) AuNPs, (**d**, **e**, **f**) AuNS HEPES, (**g**, **h**, **i**) AuNS EPPS and (**j**, **k**, **l**) AuNS MOPS at gold concentrations of 200 µM. (****) indicate groups that are significantly different with *p* < 0.0001 (one-way ANOVA). Values in columns represent mean ± standard deviation of three different batches of nanoconstructs

**Fig. 7:**
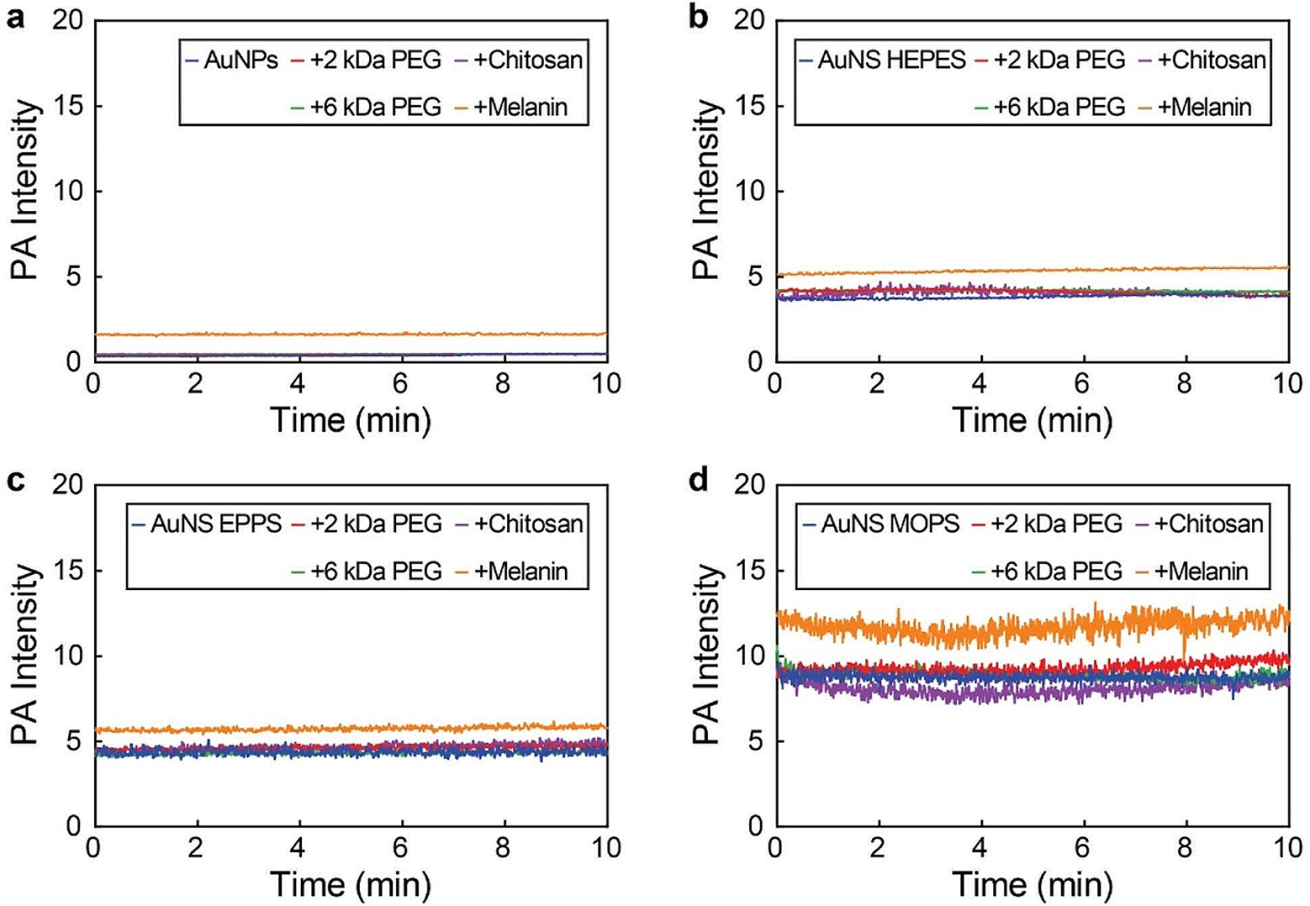
PA intensity variations of AuNPs and AuNS over time in polyethylene tubes. PA intensity of (**a**) AuNPs, (**b**) AuNS HEPES, (**c**) AuNS EPPS, and (**d**) AuNS MOPS (200 µM gold) over time under continuous irradiation

Regarding the impact of functionalization, surface modification with 2 kDa PEG, 6 kDa PEG, or chitosan had little effect on the PA response of the gold nanoconstructs, as their PA spectra remained fairly unaltered (Fig. 6, Figure S9). Conjugation with melanin, however, improved their PA responses between 1.2 and 4.5-fold. These enhancements were ascribed to the inherent photothermal characteristics of the melanin shell, which provided additional light-heat conversion. Notably, all gold nanoconstructs could be continually irradiated over 10 min without losing PA signal generation capacity (Fig. 7, Table S7), which demonstrated the photostability and durability of the nanoprobes.

Because the gold nanoconstructs are intended to be used as imaging probes, we studied their safety in vitro. The cytotoxicity of AuNPs and AuNS was studied with human embryonic kidney (HEK) 293 cells, a cell line widely used in toxicological studies [56–59]. We tested the cell inhibition under a wide range of nanoconstruct concentrations (from 0 to 200 µM). As shown in Figure S10, AuNPs, AuNS HEPES and AuNS MOPS (and their different nanoconstructs) did not induce statistically significant cytotoxicity after 24 h. Bare AuNS EPPS did induce cell death, but only at high gold concentrations (at 100 µM and above). Nevertheless, the cytotoxicity of AuNS EPPS was minimized by functionalizing the nanoparticles with the different ligands. The biocompatibility of the functionalized gold nanoconstructs observed in our in vitro experiments was consistent with previous studies. For example, it is worth noting that Auroshell, a gold nanocolloid functionalized with PEG and used as photothermal agent, has been successfully tested in three clinical studies [21]. Moreover, the other two ligands investigated in our study, chitosan and melanin, have been extensively explored for (pre)clinical pharmaceutics development and are widely regarded as safe [42, 46, 60]. To further understand the behavior of the melanin-coated AuNS in biological media, we studied their stability in 10% fetal bovine serum (FBS). Upon mixing the nanoparticles with the biological medium, the hydrodynamic radii of the particles increased (Figure S11) because of the absorption of proteins on the nanoparticle surface (a very well-known phenomenon known as opsonization). Nevertheless, after this initial protein absorption, the hydrodynamic diameter of the nanoconstructs remained stable for over 48 h, corroborating their colloidal stability in complex biological media.

**Fig. 8:**
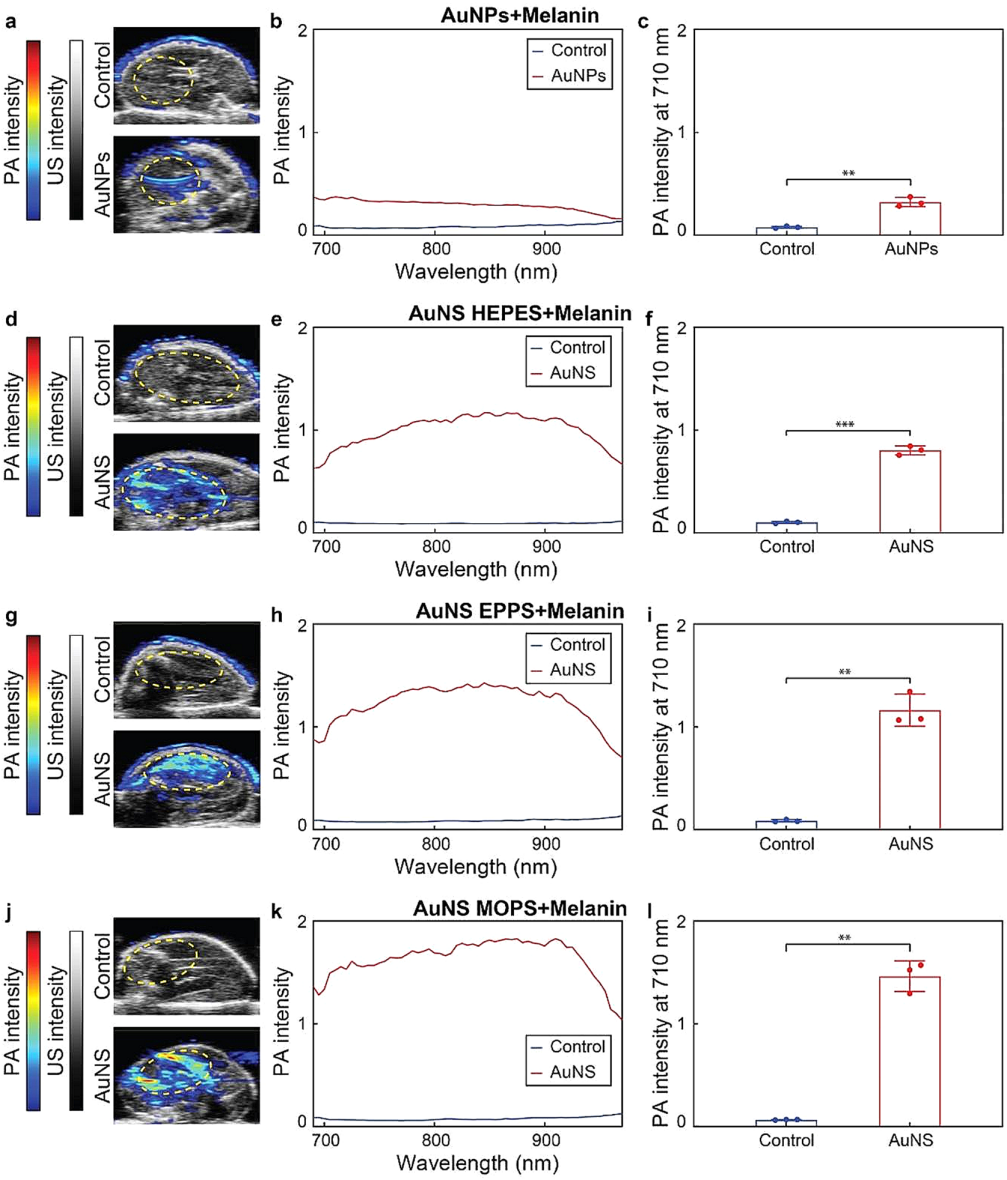
Ex vivo PA-US imaging of melanin-coated AuNPs and AuNS. PA-US images, PA spectra and PA intensities at 710 nm of melanin-coated (**a**, **b**, **c**) AuNPs, (**d**, **e**, **f**) AuNS HEPES, (**g**, **h**, **i**) AuNS EPPS and (**j**, **k**, **l**) AuNS MOPS intramuscularly injected (50 µL, 0 or 200 µM gold) in the legs of deceased mice. The color in the PA-US images represent the PA intensity at 710 nm. (**) and (***) indicate groups that are significantly different with *p* < 0.01 and *p* < 0.001, respectively (independent two-sample t-test). Values in columns represent mean ± standard deviation of three different batches of nanoconstructs. Yellow dashed areas in (a, d, g, j) indicate the regions of interest used for the spectral acquisition

Lastly, encouraged by the remarkable PA responses of the AuNS in gelatin phantoms, we carried out ex vivo PA imaging with the nanoparticles in mouse cadavers. The deceased mice were repurposed from a previous experiment, and therefore, no additional animals were sacrificed. The imaging was performed with the melanin-coated AuNPs and AuNS because of their low cytotoxicity and strong PA responses (Fig. 8, Figure S12). The nanoconstructs (200 µM, 50 µL) were intramuscularly injected into the legs and imaged with the PA system in the range from 680 to 970 nm. Compared to the control leg, low but visible PA signals appeared after the injection of melanin-coated AuNPs. Notably, strong signals were recorded in the legs after injecting the melanin-coated AuNS. Consistent with our previous results in gelatin phantoms, the nanoconstructs made of AuNS MOPS provided the strongest PA signals, as a result of their heterogenous morphologies, which displayed higher light absorption efficiencies. In this study, we have focused on the direct effects of the ligand shell and core on the photoacoustic imaging capabilities of the nanoparticles. It is worth noting, however, that shell and core features may also influence the pharmacokinetics and biodistribution of the nanoconstructs, hence, potentially affecting (indirectly) their imaging performance.

Overall, these results highlight the importance of the core structure and the surface functionalization on the performance of gold nanoconstructs for PA imaging. Among the different nanoconstructs tested, the melanin-coated AuNS MOPS performed better both in vitro and ex vivo. These nanoconstructs benefited from the strong PA imaging characteristics of the gold core (obtained with MOPS) and the melanin shell, which were preserved in biological environment. These results pave the way for future in vivo studies, where gold nanoprobes may be used for different diagnostic applications, including sentinel lymph node mapping, intraoperative imaging and tumor-associated macrophage imaging.

## Conclusions

In summary, we identified key structural features that determine the PA imaging performance of gold nanoprobes. Through an extensive study, in which the nanoparticle core morphology and ligand shell composition were systematically varied, we determined that AuNS cores - particularly those synthesized with MOPS buffer - present exceptionally high PA responses. AuNS MOPS benefited from their wider size and branch distributions, and greater light absorption efficiency, which contributed to greater PA signal. Furthermore, while most ligands only improved the nanoconstructs’ biocompatibility, melanin coating also enhanced PA signal generation, by a factor of 1.2 to 4.5, because of its unique photothermal characteristics. Notably, the strong signal generation of the gold nanoconstructs was preserved in biological environments, as demonstrated by ex vivo experiments in deceased mice. Together, these results highlight the benefits of the rational design of nanoconstructs composed of gold cores and polymer shells for high-performance PA probe development.

## Materials and methods

### Materials

4-(2-hydroxyethyl)piperazine-1-ethanesulfonic acid (HEPES), 3-(N-morpholino)propanesulf-onic acid (MOPS), Tris-(hydroxymethyl)aminomethane (TRIS) and sodium hydroxide (NaOH) were purchased from Carl Roth (Germany). 4-(2-hydroxyethyl)-1-piperazinepropa-nesulfonic acid (EPPS), sodium citrate dihydrate, 2 kDa and 6 kDa poly(ethylene glycol) methyl ether thiol (SH-PEG), chitosan (50–190 kDa), dopamine hydrochloride, and gold (III) chloride trihydrate (HAuCl4 3H_2_O) were purchased from Sigma-Aldrich (USA). XTT-based cell proliferation kit was purchased from Biological Industries (Israel). Deionized (DI) water was produced by a PURELAB flex 2 device (ELGA LabWater, Germany) and used for all experiments. All reagents were of appropriate analytical grade.

### Synthesis of AuNPs and AuNS

AuNPs were synthesized following a classic seed-mediated method. Briefly, the gold seeds were synthesized by adding 1 mL of HAuCl_4_ (25 mM) solution to 150 mL of sodium citrate (2.2 mM) solution under vigorous stirring at 100 °C. After 30 min, the seed solution was cooled down to 90 °C. 1 mL of HAuCl_4_ (25 mM) was added to the seed solution and stirred for 30 min to grow the nanoparticles. After repeating this process twice, 55 mL of the gold nanoparticle solution was diluted by adding 53 mL of water and 2 mL of sodium citrate (60 mM) solution. The resulting solution was heated up to 90 °C, and then 1 mL of HAuCl_4_ (25 mM) was added. This process was repeated four more times. The AuNPs were washed with DI water by centrifugation (7000 rpm for 10 min).

AuNS were synthesized following a seedless method. Briefly, 0.75 mL of HEPES (1 M, pH 7.4), 1.5 mL of EPPS (1 M, pH 7.3) or 2 mL of MOPS (1 M, pH 7.0) were diluted in DI water for a final volume of 9.8 mL, and then 0.2 mL of HAuCl_4_ (10 mM) was added. The solutions were vigorously stirred for 30 s and left without disturbing for 2 h. The AuNS were washed with DI water by centrifugation (7000 rpm for 10 min).

### PEG functionalization (PEGylation of AuNPs and AuNS)

1 mL of AuNPs or AuNS (1 mM gold) was added to 3 mL of SH-PEG (2 kDa or 6 kDa) solution with a final SH-PEG: gold molar ratio of 1, 2 or 4. The solution was stirred at room temperature for 4 h and washed with DI water by centrifugation (7000 rpm for 10 min).

### Chitosan functionalization

0.5 mL of AuNPs or AuNS (0.2 mg/mL gold) was added to 9.5 mL of chitosan solution with a final chitosan : gold mass ratio of 20, 30 or 50. The solution was stirred at room temperature for 4 h and washed with DI water by centrifugation (7000 rpm for 10 min).

### Melanin functionalization

0.5 mL of AuNPs or AuNS (1 mM gold) was added to 9.49 mL of dopamine solution with a final dopamine : gold molar ratio of 1, 5 or 10. The solution was stirred at room temperature for 5 min and 0.01 mL of Tris solution (187 mg/mL) was added. The resulting solution was stirred at room temperature for 4 h and washed with DI water by centrifugation (7000 rpm for 10 min).

### Characterizations

The extinction spectra of AuNPs and AuNS were measured with an Infinite M200 Pro microplate reader (Tecan, Switzerland). The reader was set in absorbance scan mode with a scanning interval of 2 nm. The hydrodynamic size and zeta potential were measured with a Nanosizer ZS (Malvern, UK). The gold concentration was determined by inductively coupled plasma mass spectrometry (ICP-MS) with an Agilent 8900 (Agilent, USA). Before ICP-MS analysis, the AuNPs and AuNS were digested with a mixture of HCl and HNO_3_, which has been previously used to digest noble metal nanoconstructs [61, 62]. The morphology was imaged with a 100-kV transmission electron microscope system (Hitachi, Japan). The Fourier-transform infrared (FTIR) spectra were measured with a Spectrum 3 FTIR spectrometer (PerkinElmer, USA). Before FTIR analysis, the AuNPs and AuNS were lyophilized with an Alpha 2–4 LD PLUS (Martin Chirist, Germany) [63].

### Photoacoustic (PA) imaging

The PA imaging performances of AuNPs and AuNS were evaluated in solidified 10% (w/v) gelatin phantoms (Figure S7a). 0.3 mL of AuNPs or AuNS solutions (200 µM gold, 2% (w/v) gelatin) was added into the sample holes on the phantom and solidified at 4 °C for 5 h. The VevoLAZR system (Visualsonics, Canada) was used to image the sample-bearing phantoms with excitation wavelengths from 680 to 970 nm. The PA stabilities of AuNPs and AuNS were performed in low-density polyethylene tubes (Figure S7b). Hence, 0.05 mL of AuNP or AuNS solutions (200 µM gold) were introduced in the polyethylene tubes and imaged with excitation wavelengths ranging from 680 to 970 nm. The PA intensities at 710 nm displayed in Figs. 4, 6 and 8 were determined from the spectral acquisition mode, which ranged from 680 to 970 nm. Only in the PA stability studies (Fig. 7), the PA intensities at 710 nm were obtained from single wavelength recordings, which were continuously acquired for 10 min.

### Cytotoxicity of AuNPs and AuNS

Human embryonic kidney (HEK) 293 cells were used for the in vitro cytotoxicity test of AuNPs and AuNS. HEK 293 cells were cultured in a 96-well plate at a density of 10,000 cells/well for 12 h in minimum essential medium (MEM) supplemented with 10% FBS and 1% penicillin/streptomycin at 37 °C with 5% CO_2_. AuNPs or AuNS with different concentrations (0, 3.13, 6.25, 12.5, 25, 50, 100 and 200 µM gold) were then cultured with HEK 293 for 24 h. The proliferation test was carried out under the standard tetrazolium chloride (XTT) assay.

### Analytical method

The photoacoustic intensity that is generated by a small plasmonic nanoparticle as part of an ensemble can be described in terms of a point source model [64]. In this case, the total photoacoustic intensity can be approximated by the following Eqs. [41, 55]:

1 $${S_{PA}}{\mkern 1mu} \,\alpha {\mkern 1mu} {W_0}{\mkern 1mu} {{{\sigma _a}} \over {{\sigma _e}}}{\mkern 1mu} \left( {1 - exp\left( { - {\sigma _e}{L_{{C_n}}}} \right)} \right)\, = \,A\left( {1 - exp\left( { - Bc} \right)} \right)$$

Here, ${W_0}$ is the power of the laser at the sample entrance, ${\sigma _a}\,\left( {{\sigma _e}} \right)$ is the absorption (extinction) cross-section of the nanoparticle, ${c_n}\,\left( c \right)$ is the nanoparticle number (molar) concentration, $L$ is the total sample length, $A$ and $B$ are appropriate coefficients. In the limit of small concentrations, the total photoacoustic intensity is a linear function of the concentration ${S_{PA}}\,\mathrel{\mathop{\kern0pt\longrightarrow}\limits_{c \to 0}} \,\left( {AB} \right)c$. The measured photoacoustic intensity was fitted using Eq. (1) as a function of molar concentration for different excitation wavelengths. The resulting spectra of the $A$ and $B$ coefficients allow the absorption cross-section per nanoparticle volume to be reconstructed as a function of wavelength:

2 $${{{\sigma _a}} \over {{V_p}}}\, = \,{{AB} \over {{W_0}}}\,{\left( {{{L{{10}^{ - 3}}MW} \over p}} \right)^{ - 1}}$$

Here $MW$ and $\rho$ are the molar mass and the mass density of the nanoparticle, respectively. In deriving Eq. (2), we have assumed that the proportionality coefficient in the first part of (1) is both wavelength and nanoparticle type independent. The ratio of PA intensity between different types of nanoparticles is determined by the ratio of their absorption cross-section per nanoparticle volume.

### Ex vivo PA imaging

As controls, the legs of mouse cadavers were first imaged under the VevoLAZR system with excitation wavelengths ranging from 680 to 970 nm. Next, 50 µL of melanin-coated AuNPs (200 µM gold) or AuNS (HEPES, EPPS or MOPS, 200 µM gold) was intramuscularly injected into the legs and imaged with the same settings.

### Statistical analysis

Three different batches of gold nanoconstructs were prepared for each core and shell type. All values are presented as mean ± standard deviation. Statistical analyses were performed using GraphPad Prism 8.

### Electronic supplementary material

Below is the link to the electronic supplementary material.

**Supplementary Material 1**: **Figure S1**. Distributions of root width of AuNS HEPES, EPPS and MOPS. **Table S1**. Morphological features of AuNS HEPES, EPPS and MOPS. **Table S2**. Branch characteristics of AuNS HEPES. **Table S3**. Branch characteristics of AuNS EPPS. **Table S4**. Branch characteristics of AuNS MOPS. **Figure S2**. Functionalization of AuNPs with the different ligands. (a, e, i, m) Hydrodynamic diameter (HD), (b, f, j, n) polydispersity index (PDI), (c, g, k, o) zeta potential and (d, h, l, p) extinction spectra of AuNPs functionalized with 2 kDa PEG, 6 kDa PEG, chitosan and melanin. **Figure S3**. Functionalization of AuNS HEPES with the different ligands. (a, e, i, m) Hydrodynamic diameter (HD), (b, f, j, n) polydispersity index (PDI), (c, g, k, o) zeta potential and (d, h, l, p) extinction spectra of AuNS HEPES functionalized with 2 kDa PEG, 6 kDa PEG, chitosan and melanin. **Figure S4**. TEM micrographs of AuNPs (a, f) before and after functionalization with (b, g) 2 kDa PEG, (c, h) 6 kDa PEG, (d, i) chitosan and (e, j) melanin. **Figure S5**. TEM micrographs of AuNS HEPES. TEM micrographs of AuNS (a, f) before and after functionalization with (b, g) 2 kDa PEG, (c, h) 6 kDa PEG, (d, i) chitosan and (e, j) melanin. **Table S5**. Optimal functionalization conditions and characterization of AuNPs. **Table S6**. Optimal functionalization conditions and characterization of AuNS HEPES. **Figure S6**. FTIR spectra of functionalized AuNPs and AuNS with (a) 2 kDa PEG, (b) 6 kDA PEG, (c) chitosan and (d) melanin. **Figure S7**. Phantoms used for PA imaging characterization. (a) Homemade gelatin phantom, and (b) low density polyethylene tubes used for the photoacoustic experiments before and after nanoparticle loading. **Figure S8**. Signal-to-noise ratios (SNRs) of PA intensities of AuNPs and AuNS. SNRs of PA intensities at 710 nm of (a) AuNPs, (b) AuNS HEPES, (c) AuNS EPPS, and (d) AuNS MOPS at different gold concentrations (from 0 to 200 mM). **Figure S9**. SNRs of PA intensities of functionalized AuNPs and AuNS. SNRs of PA intensities at 710 nm of functionalized (a) AuNPs, (b) AuNS HEPES, (c) AuNS EPPS, and (d) AuNS MOPS at gold concentrations of 200 mM. **Table S7**. Variation coefficients of PA intensities at 710 nm of (a) AuNPs, (b) AuNS HEPES, (c) AuNS EPPS and (d) AuNS MOPS at gold concentrations of 200 mM gold in polyethylene tubes. **Figure S10**. Cytotoxicity of AuNPs and AuNS. Cell viability of HEK 293 cells after 24 hours of incubation with (a) AuNPs, (b) AuNS HEPES, (c) AuNS EPPS and (d) AuNS MOPS (from 0 to 200 mM of gold). **Figure S11**. Stability of melanin-coated AuNS in 10 % fetal bovine serum (FBS). Hydrodynamic diameter (HD) of (a) melanin-coated AuNS HEPES, (b) EPPS and (c) MOPS in 10 % FBS at different incubation times. **Figure S12**. SNRs of PA intensities of melanin-coated AuNPs and AuNS in ex vivo imaging. SNRs of PA intensities at 710 nm of melanin-coated (a) AuNPs, (b) AuNS HEPES, (c) AuNS EPPS and (d) AuNS MOPS intramuscularly injected (50 mL, 0 or 200 mM gold) in the legs of deceased mice

## Data Availability

No datasets were generated or analysed during the current study.

## Declarations

### Supporting Information

The following files are available free of charge. Distributions of root width of AuNS HEPES, EPPS and MOPS; morphological features of AuNS HEPES, EPPS and MOPS; branch characteristics of AuNS HEPES; branch characteristics of AuNS EPPS; branch characteristics of AuNS MOPS; functionalization of AuNPs with the different ligands; functionalization of AuNS HEPES with the different ligands; TEM micrographs of AuNPs; TEM micrographs of AuNS HEPES; optimal functionalization conditions and characterization of AuNPs; optimal functionalization conditions and characterization of AuNS HEPES; FTIR spectra of functionalized AuNPs and AuNS; PA intensity of melanin-coated AuNPs and AuNS; cytotoxicity of AuNPs and AuNS; and stability of melanin-coated AuNS in 10% fetal bovine serum (PDF).
