# Supplementary material for: Nanoscale engineering of gold nanostars for enhanced photoacoustic imaging
Source: J Nanobiotechnology. 2024 Mar 16;22:115. doi: 10.1186/s12951-024-02379-7 (PMC10943878; doi:10.1186/s12951-024-02379-7)
Supplement: Supplementary file 1 — Supplementary Material 1: Figure S1. Distributions of root width of AuNS HEPES, EPPS and MOPS. Table S1. Morphological features of AuNS HEPES, EPPS and MOPS. Table S2. Branch characteristics of AuNS HEPES. Table S3. Branch characteristics of AuNS EPPS. Table S4. Branch characteristics of AuNS MOPS. Figure S2. Functionalization of AuNPs with the different ligands. (a, e, i, m) Hydrodynamic diameter (HD), (b, f, j, n) polydispersity index (PDI), (c, g, k, o) zeta potential and (d, h, l, p) extinction spectra of AuNPs functionalized with 2 kDa PEG, 6 kDa PEG, chitosan and melanin. Figure S3. Functionalization of AuNS HEPES with the different ligands. (a, e, i, m) Hydrodynamic diameter (HD), (b, f, j, n) polydispersity index (PDI), (c, g, k, o) zeta potential and (d, h, l, p) extinction spectra of AuNS HEPES functionalized with 2 kDa PEG, 6 kDa PEG, chitosan and melanin. Figure S4. TEM micrographs of AuNPs (a, f) before and after functionalization with (b, g) 2 kDa PEG, (c, h) 6 kDa PEG, (d, i) chitosan and (e, j) melanin. Figure S5. TEM micrographs of AuNS HEPES. TEM micrographs of AuNS (a, f) before and after functionalization with (b, g) 2 kDa PEG, (c, h) 6 kDa PEG, (d, i) chitosan and (e, j) melanin. Table S5. Optimal functionalization conditions and characterization of AuNPs. Table S6. Optimal functionalization conditions and characterization of AuNS HEPES. Figure S6. FTIR spectra of functionalized AuNPs and AuNS with (a) 2 kDa PEG, (b) 6 kDA PEG, (c) chitosan and (d) melanin. Figure S7. Phantoms used for PA imaging characterization. (a) Homemade gelatin phantom, and (b) low density polyethylene tubes used for the photoacoustic experiments before and after nanoparticle loading. Figure S8. Signal-to-noise ratios (SNRs) of PA intensities of AuNPs and AuNS. SNRs of PA intensities at 710 nm of (a) AuNPs, (b) AuNS HEPES, (c) AuNS EPPS, and (d) AuNS MOPS at different gold concentrations (from 0 to 200 mM). Figure S9. SNRs of PA intensities of functionalized AuNPs and AuN [file 12951_2024_2379_MOESM1_ESM.docx]

**- Supporting Information -**

**Nanoscale Engineering of Gold Nanostars for Enhanced Photoacoustic Imaging**

Rui Zhang,^1^ Sven Thoröe-Boveleth,^2^ Dmitry N. Chigrin,^3,4^ Fabian Kiessling,^1^ Twan Lammers,^1^ and Roger M. Pallares^1,*^

^1^Institute for Experimental Molecular Imaging, RWTH Aachen University Hospital, Aachen 52074, Germany

^2^Institute for Occupational, Social and Environmental Medicine, Medical Faculty, RWTH Aachen University, Aachen 52074, Germany

^3^Institue of Physics (1A), RWTH Aachen University, 52056 Aachen, Germany

^4^DWI − Leibniz Institute for Interactive Materials, 52076 Aachen, Germany

^*^Corresponding author: [rmoltopallar@ukaachen.de](mailto:rmoltopallar@ukaachen.de)

**Table of contents**

[Figure S1. Distributions of root width of AuNS HEPES, EPPS and MOPS. S3](https://ukaachen-my.sharepoint.com/personal/rzhang_ukaachen_de/Documents/3%20project-AuNSs%20in%20PAI/manuscript/SI%20-%20Nanoscale%20Engineering%20of%20Gold%20Nanostars%20for%20Enhanced%20Photoacoustic%20Imaging%20-%20JNB.docx#_Toc158208548)

[Table S1. Morphological features of AuNS HEPES, EPPS and MOPS. S4](https://ukaachen-my.sharepoint.com/personal/rzhang_ukaachen_de/Documents/3%20project-AuNSs%20in%20PAI/manuscript/SI%20-%20Nanoscale%20Engineering%20of%20Gold%20Nanostars%20for%20Enhanced%20Photoacoustic%20Imaging%20-%20JNB.docx#_Toc158208549)

[Table S2. Branch characteristics of AuNS HEPES. S4](https://ukaachen-my.sharepoint.com/personal/rzhang_ukaachen_de/Documents/3%20project-AuNSs%20in%20PAI/manuscript/SI%20-%20Nanoscale%20Engineering%20of%20Gold%20Nanostars%20for%20Enhanced%20Photoacoustic%20Imaging%20-%20JNB.docx#_Toc158208550)

[Table S3. Branch characteristics of AuNS EPPS. S4](https://ukaachen-my.sharepoint.com/personal/rzhang_ukaachen_de/Documents/3%20project-AuNSs%20in%20PAI/manuscript/SI%20-%20Nanoscale%20Engineering%20of%20Gold%20Nanostars%20for%20Enhanced%20Photoacoustic%20Imaging%20-%20JNB.docx#_Toc158208551)

[Table S4. Branch characteristics of AuNS MOPS. S4](https://ukaachen-my.sharepoint.com/personal/rzhang_ukaachen_de/Documents/3%20project-AuNSs%20in%20PAI/manuscript/SI%20-%20Nanoscale%20Engineering%20of%20Gold%20Nanostars%20for%20Enhanced%20Photoacoustic%20Imaging%20-%20JNB.docx#_Toc158208552)

[Figure S2. Functionalization of AuNPs with the different ligands. S5](https://ukaachen-my.sharepoint.com/personal/rzhang_ukaachen_de/Documents/3%20project-AuNSs%20in%20PAI/manuscript/SI%20-%20Nanoscale%20Engineering%20of%20Gold%20Nanostars%20for%20Enhanced%20Photoacoustic%20Imaging%20-%20JNB.docx#_Toc158208553)

[Figure S3. Functionalization of AuNS HEPES with the different ligands. S6](https://ukaachen-my.sharepoint.com/personal/rzhang_ukaachen_de/Documents/3%20project-AuNSs%20in%20PAI/manuscript/SI%20-%20Nanoscale%20Engineering%20of%20Gold%20Nanostars%20for%20Enhanced%20Photoacoustic%20Imaging%20-%20JNB.docx#_Toc158208554)

[Figure S4. TEM micrographs of AuNPs. S7](https://ukaachen-my.sharepoint.com/personal/rzhang_ukaachen_de/Documents/3%20project-AuNSs%20in%20PAI/manuscript/SI%20-%20Nanoscale%20Engineering%20of%20Gold%20Nanostars%20for%20Enhanced%20Photoacoustic%20Imaging%20-%20JNB.docx#_Toc158208555)

[Figure S5. TEM micrographs of AuNS HEPES. S8](https://ukaachen-my.sharepoint.com/personal/rzhang_ukaachen_de/Documents/3%20project-AuNSs%20in%20PAI/manuscript/SI%20-%20Nanoscale%20Engineering%20of%20Gold%20Nanostars%20for%20Enhanced%20Photoacoustic%20Imaging%20-%20JNB.docx#_Toc158208556)

[Table S5. Optimal functionalization conditions and characterization of AuNPs. S9](https://ukaachen-my.sharepoint.com/personal/rzhang_ukaachen_de/Documents/3%20project-AuNSs%20in%20PAI/manuscript/SI%20-%20Nanoscale%20Engineering%20of%20Gold%20Nanostars%20for%20Enhanced%20Photoacoustic%20Imaging%20-%20JNB.docx#_Toc158208557)

[Table S6. Optimal functionalization conditions and characterization of AuNS HEPES. S9](https://ukaachen-my.sharepoint.com/personal/rzhang_ukaachen_de/Documents/3%20project-AuNSs%20in%20PAI/manuscript/SI%20-%20Nanoscale%20Engineering%20of%20Gold%20Nanostars%20for%20Enhanced%20Photoacoustic%20Imaging%20-%20JNB.docx#_Toc158208558)

[Figure S6. FTIR spectra of functionalized AuNPs and AuNS. S10](https://ukaachen-my.sharepoint.com/personal/rzhang_ukaachen_de/Documents/3%20project-AuNSs%20in%20PAI/manuscript/SI%20-%20Nanoscale%20Engineering%20of%20Gold%20Nanostars%20for%20Enhanced%20Photoacoustic%20Imaging%20-%20JNB.docx#_Toc158208559)

[Figure S7. Phantoms used for PA imaging characterization. S11](https://ukaachen-my.sharepoint.com/personal/rzhang_ukaachen_de/Documents/3%20project-AuNSs%20in%20PAI/manuscript/SI%20-%20Nanoscale%20Engineering%20of%20Gold%20Nanostars%20for%20Enhanced%20Photoacoustic%20Imaging%20-%20JNB.docx#_Toc158208560)

[Figure S8. SNRs of PA intensities of AuNPs and AuNS. S12](https://ukaachen-my.sharepoint.com/personal/rzhang_ukaachen_de/Documents/3%20project-AuNSs%20in%20PAI/manuscript/SI%20-%20Nanoscale%20Engineering%20of%20Gold%20Nanostars%20for%20Enhanced%20Photoacoustic%20Imaging%20-%20JNB.docx#_Toc158208561)

[Figure S9. SNRs of PA intensities of functionalized AuNPs and AuNS. S13](https://ukaachen-my.sharepoint.com/personal/rzhang_ukaachen_de/Documents/3%20project-AuNSs%20in%20PAI/manuscript/SI%20-%20Nanoscale%20Engineering%20of%20Gold%20Nanostars%20for%20Enhanced%20Photoacoustic%20Imaging%20-%20JNB.docx#_Toc158208562)

[Table S7. Variations of PA intensities. S14](https://ukaachen-my.sharepoint.com/personal/rzhang_ukaachen_de/Documents/3%20project-AuNSs%20in%20PAI/manuscript/SI%20-%20Nanoscale%20Engineering%20of%20Gold%20Nanostars%20for%20Enhanced%20Photoacoustic%20Imaging%20-%20JNB.docx#_Toc158208563)

[Figure S10. Cytotoxicity of AuNPs and AuNS. S15](https://ukaachen-my.sharepoint.com/personal/rzhang_ukaachen_de/Documents/3%20project-AuNSs%20in%20PAI/manuscript/SI%20-%20Nanoscale%20Engineering%20of%20Gold%20Nanostars%20for%20Enhanced%20Photoacoustic%20Imaging%20-%20JNB.docx#_Toc158208564)

[Figure S11. Stability of melanin-coated AuNS in 10 % fetal bovine serum (FBS). S16](https://ukaachen-my.sharepoint.com/personal/rzhang_ukaachen_de/Documents/3%20project-AuNSs%20in%20PAI/manuscript/SI%20-%20Nanoscale%20Engineering%20of%20Gold%20Nanostars%20for%20Enhanced%20Photoacoustic%20Imaging%20-%20JNB.docx#_Toc158208565)

[Figure S12. SNRs of PA intensities of melanin-coated AuNPs and AuNS in ex vivo imaging. S17](https://ukaachen-my.sharepoint.com/personal/rzhang_ukaachen_de/Documents/3%20project-AuNSs%20in%20PAI/manuscript/SI%20-%20Nanoscale%20Engineering%20of%20Gold%20Nanostars%20for%20Enhanced%20Photoacoustic%20Imaging%20-%20JNB.docx#_Toc158208566)


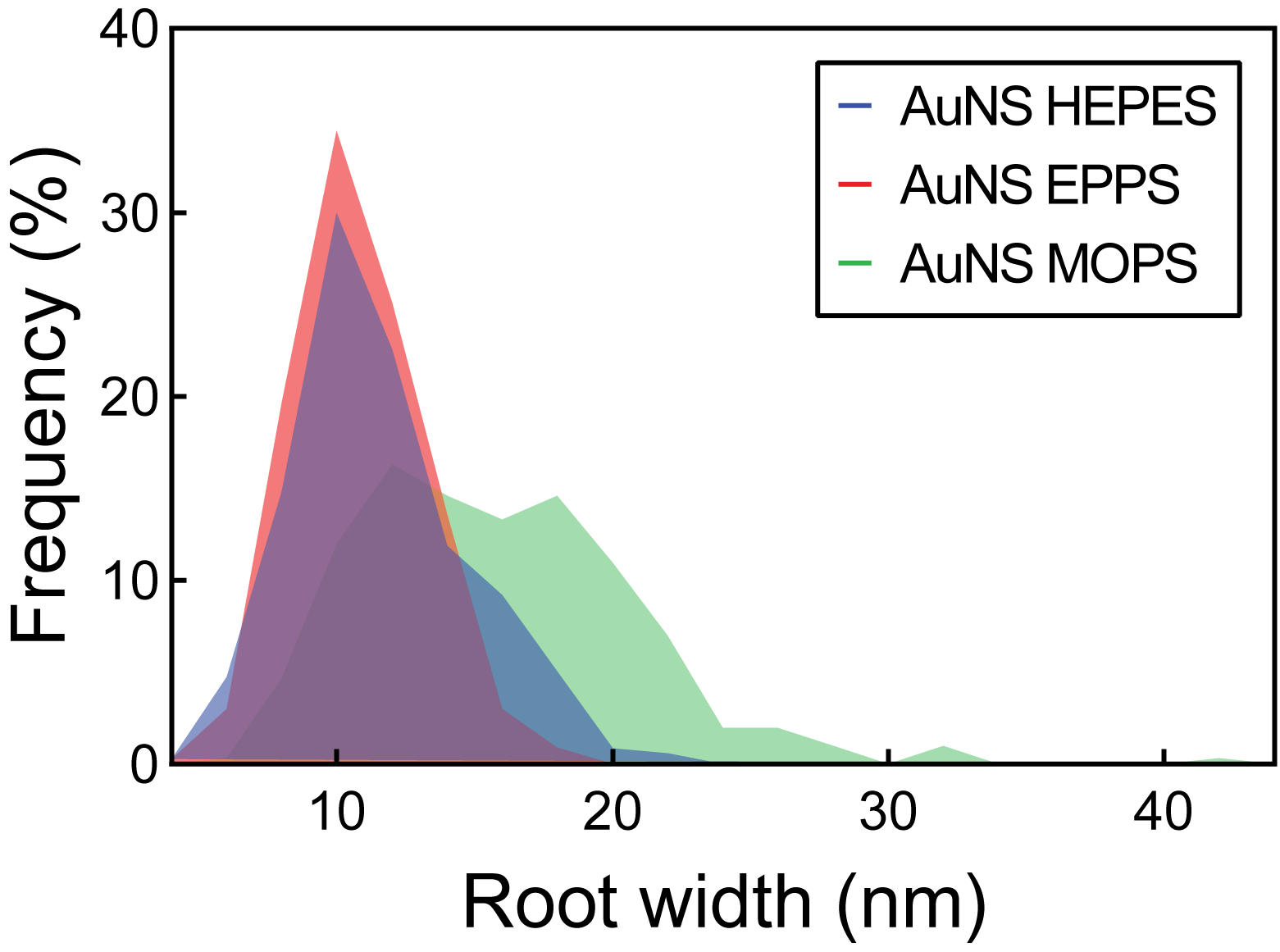


# Figure S1. Distributions of root width of AuNS HEPES, EPPS and MOPS.

| Table S1. Morphological features of AuNS HEPES, EPPS and MOPS. | | | | | | | |
| --- | --- | --- | --- | --- | --- | --- | --- |
|  | Feret Diameter (nm) | Core Size (nm) | Numbers of Branches | Length  (nm) | Middle Width  (nm) | Root Width  (nm) | Aspect Ratio |
| AuNS  HEPES | 43.5 ± 12.8 | 19.3 ± 5.5 | 3.6 ± 1.6 | 12.4 ± 1.6 | 8.6 ± 2.4 | 11.5 ± 3.1 | 1.5 ± 0.6 |
| AuNS  EPPS | 48.2 ± 14.2 | 18.0 ± 4.6 | 3.6 ± 1.3 | 13.3 ± 6.4 | 8.1 ± 2.0 | 10.8 ± 2.3 | 1.5 ± 0.7 |
| AuNS  MOPS | 48.7 ± 21.3 | 26.0 ± 10.0 | 3.2 ± 1.4 | 19.5 ± 14.6 | 11.1 ± 3.4 | 15.8 ± 5.0 | 1.7 ± 0.9 |
| 100+ particles | | | | 300+ branches | | | |

| Table S2. Branch characteristics of AuNS HEPES. | | | | | |
| --- | --- | --- | --- | --- | --- |
| Branch length distribution | Proportion | Length  (nm) | Middle Width  (nm) | Root Width  (nm) | Aspect Ratio |
| < 30 nm | 98.5 % | 12.0 ± 4.7 | 8.6 ± 2.4 | 11.4 ± 3.0 | 1.4 ± 0.5 |
| 30-60 nm | 1.5 % | 37.1 ± 5.8 | 10.8 ± 2.7 | 16.1 ± 2.1 | 3.5 ± 0.7 |
| > 60 nm | 0 | / | / | / | / |

| Table S3. Branch characteristics of AuNS EPPS. | | | | | |
| --- | --- | --- | --- | --- | --- |
| Branch length distribution | Proportion | Length  (nm) | Middle Width  (nm) | Root Width  (nm) | Aspect Ratio |
| < 30 nm | 98.2 % | 11.9 ± 5.7 | 8.0 ± 2.0 | 10.7 ± 2.3 | 1.5 ± 0.7 |
| 30-60 nm | 1.8 % | 34.5 ± 4.2 | 10.2 ± 2.3 | 13.6 ± 1.9 | 3.5 ± 0.8 |
| > 60 nm | 0 | / | / | / | / |

| Table S4. Branch characteristics of AuNS MOPS. | | | | | |
| --- | --- | --- | --- | --- | --- |
| Branch length distribution | Proportion | Length  (nm) | Middle Width  (nm) | Root Width  (nm) | Aspect Ratio |
| < 30 nm | 83.4 % | 14.1 ± 5.9 | 10.4 ± 3.2 | 14.7 ± 4.0 | 1.4 ± 0.5 |
| 30-60 nm | 13.0 % | 41.2 ± 9.2 | 14.2 ± 2.6 | 20.8 ± 4.5 | 3.0 ± 0.7 |
| > 60 nm | 3.6 % | 67.9 ± 6.4 | 15.4 ± 1.9 | 25.4 ± 4.6 | 4.5 ± 0.7 |


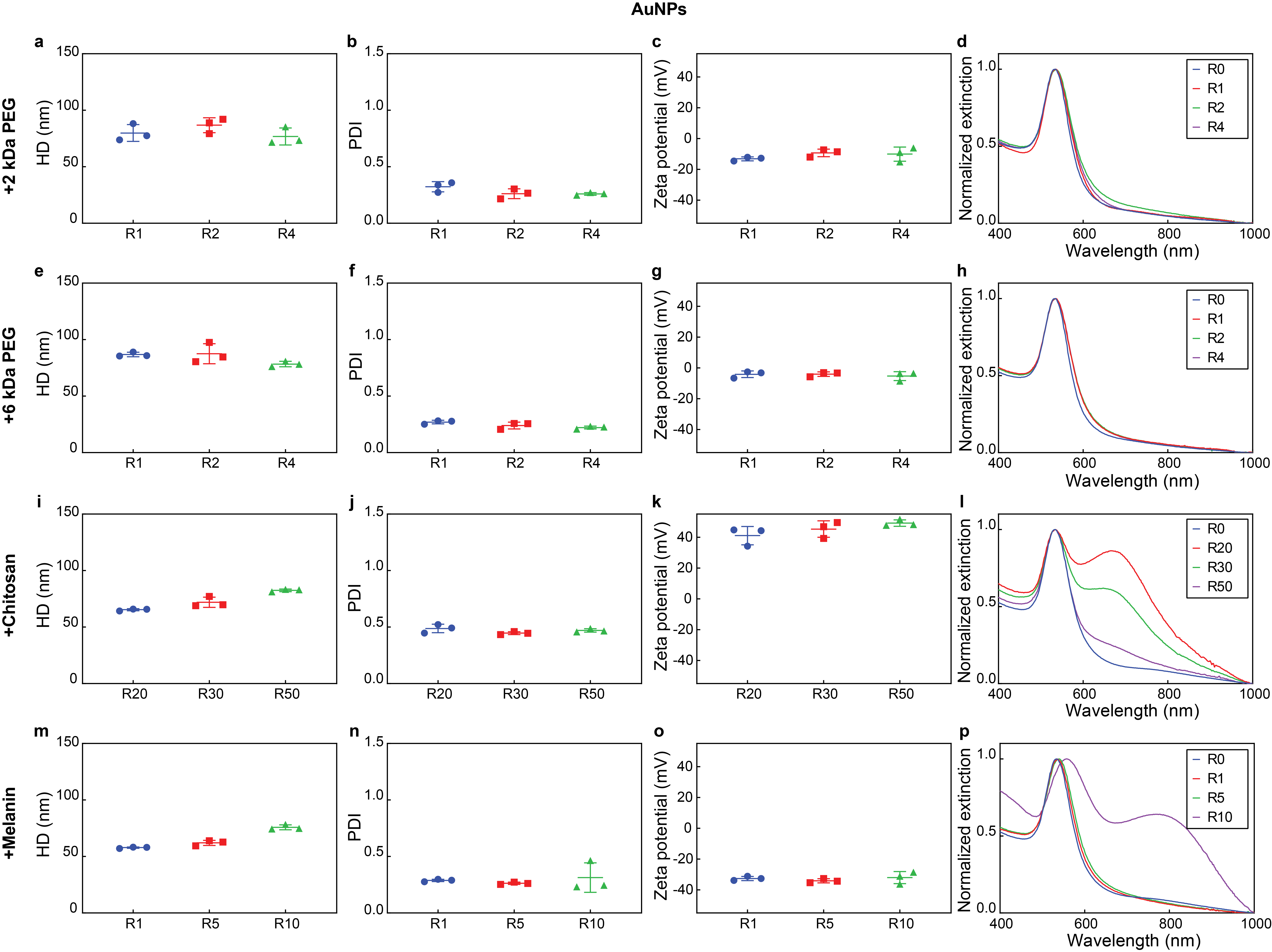


Figure S2. Functionalization of AuNPs with the different ligands. (**a, e, i, m**) Hydrodynamic diameter (HD), (**b, f, j, n**) polydispersity index (PDI), (**c, g, k, o**) zeta potential and (**d, h, l, p**) extinction spectra of AuNPs functionalized with 2 kDa PEG, 6 kDa PEG, chitosan and melanin.


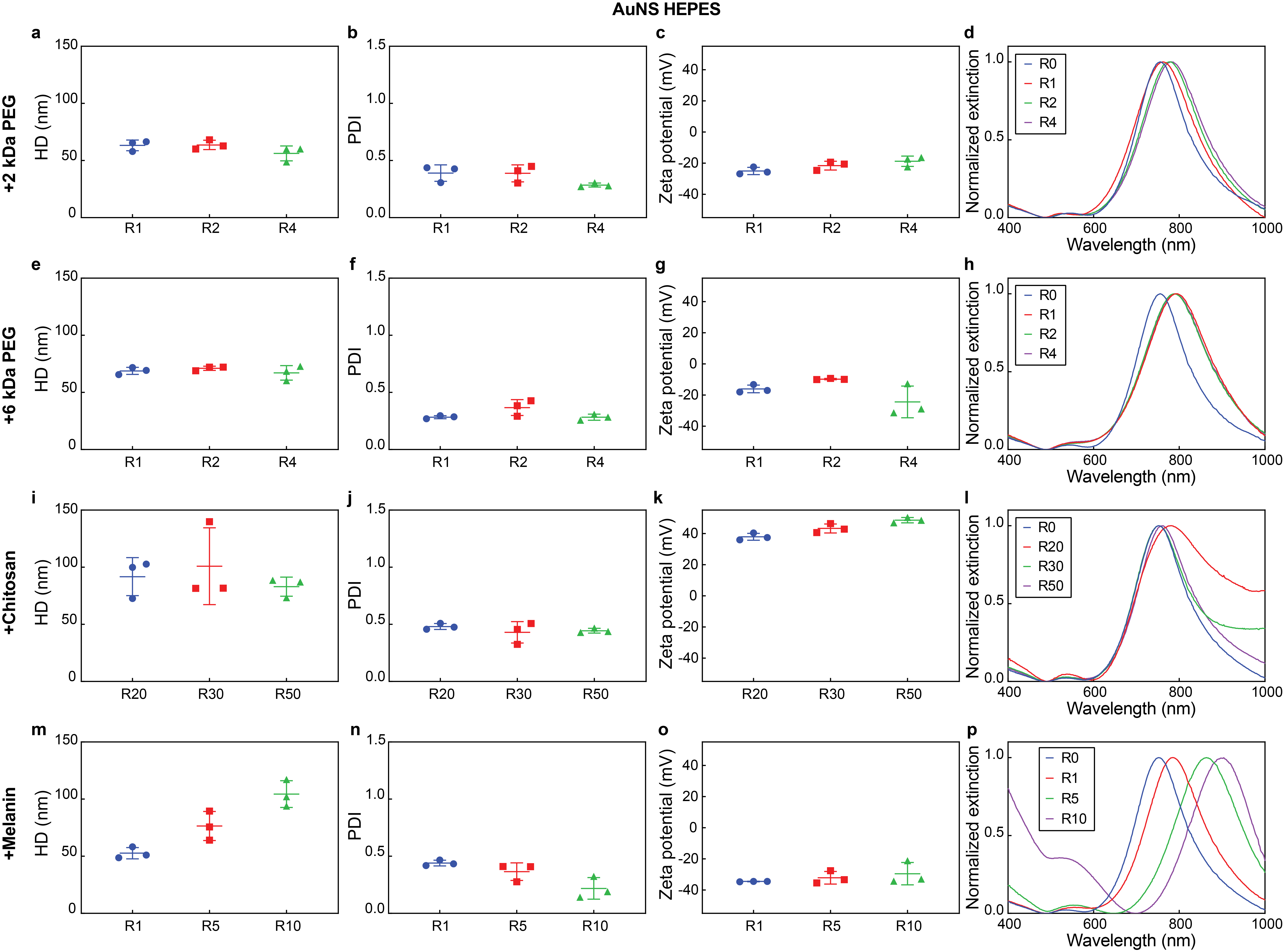


Figure S3. Functionalization of AuNS HEPES with the different ligands. (**a, e, i, m**) Hydrodynamic diameter (HD), (**b, f, j, n**) polydispersity index (PDI), (**c, g, k, o**) zeta potential and (**d, h, l, p**) extinction spectra of AuNS HEPES functionalized with 2 kDa PEG, 6 kDa PEG, chitosan and melanin.


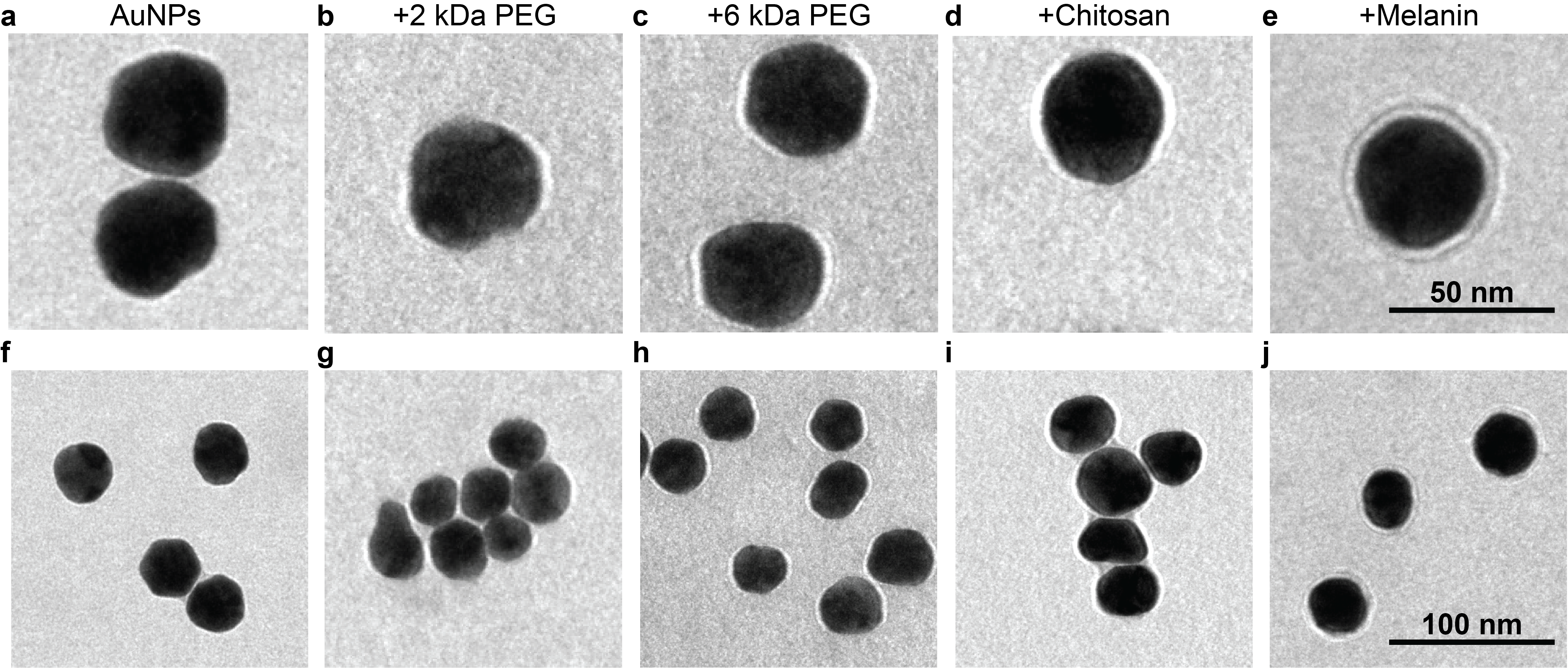


Figure S4. TEM micrographs of AuNPs. TEM micrographs of AuNPs (**a, f**) before and after functionalization with (**b, g**) 2 kDa PEG, (**c, h**) 6 kDa PEG, (**d, i**) chitosan and (**e, j**) melanin.


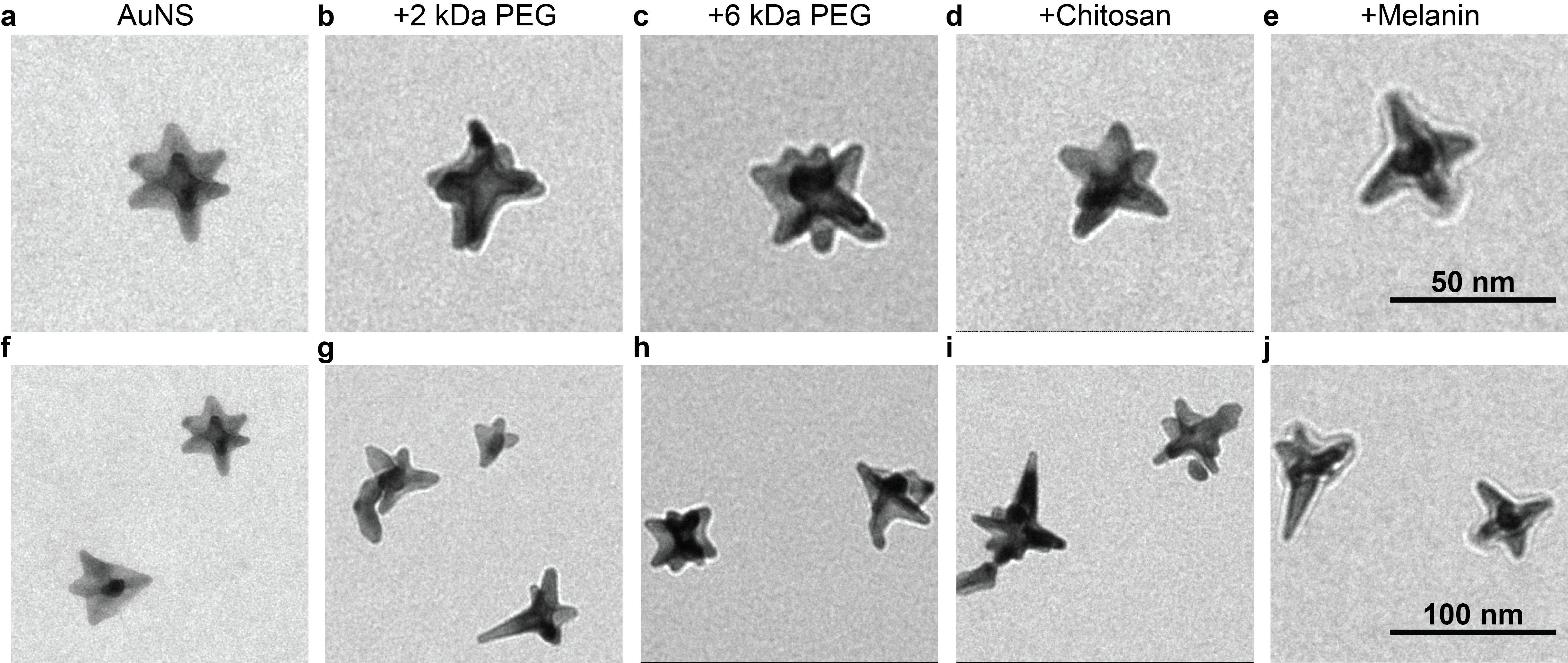


Figure S5. TEM micrographs of AuNS HEPES. TEM micrographs of AuNS (**a, f**) before and after functionalization with (**b, g**) 2 kDa PEG, (**c, h**) 6 kDa PEG, (**d, i**) chitosan and (**e, j**) melanin.

| Table S5. Optimal functionalization conditions and characterization of AuNPs. | | | | |
| --- | --- | --- | --- | --- |
|  | Ligand : Au | Hydrodynamic size (nm) | LSP band (nm) | Zeta potential (mV) |
| AuNPs | / | 50.8 ± 0.6 | 534 | -23.7 ± 0.8 |
| + 2 kDa PEG | 1(molar) | 79.9 ± 7.4 | 536 | -13.2 ± 1.3 |
| + 6 kDa PEG | 2 (molar) | 87.6 ± 8.8 | 534 | -9.3 ± 2.4 |
| + Chitosan | 50 (mass) | 82.5 ± 1.1 | 536 | 49.2 ± 2.1 |
| + Melanin | 1 (molar) | 57.9 ± 0.6 | 536 | -32.6 ± 1.3 |

| Table S6. Optimal functionalization conditions and characterization of AuNS HEPES. | | | | |
| --- | --- | --- | --- | --- |
|  | Ligand : Au | Hydrodynamic size (nm) | LSP band (nm) | Zeta potential (mV) |
| AuNS HEPES | / | 38.2 ± 0.2 | 752 | -29.5 ± 1.4 |
| + 2 kDa PEG | 1(molar) | 63.3 ± 4.7 | 762 | -25.0 ± 2.3 |
| + 6 kDa PEG | 2 (molar) | 71.1 ± 1.8 | 788 | -9.8 ± 0.4 |
| + Chitosan | 50 (mass) | 83.0 ± 8.3 | 762 | 48.5 ± 1.7 |
| + Melanin | 1 (molar) | 52.7 ± 4.9 | 786 | -34.5 ± 0.2 |


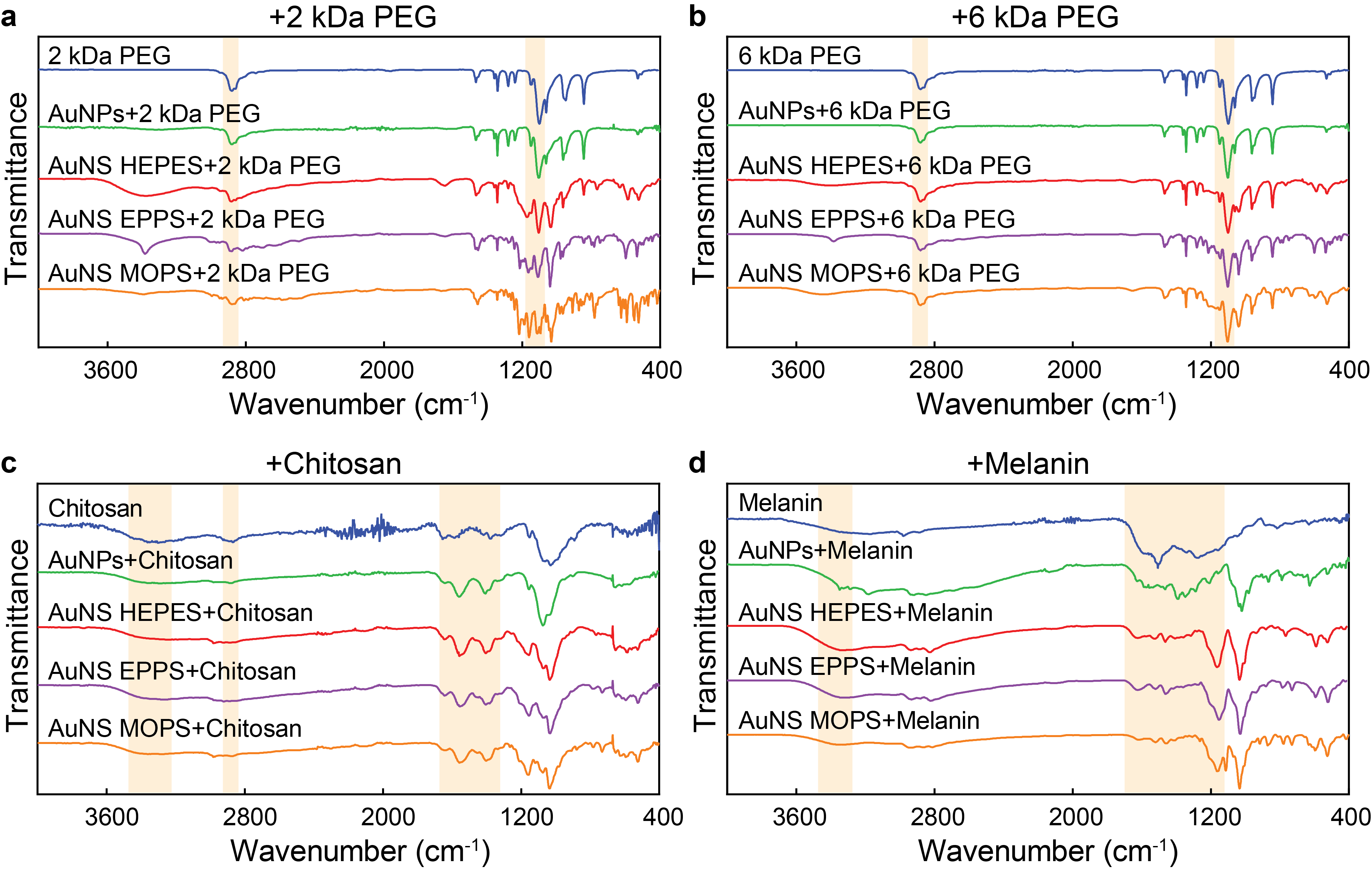


Figure S6. FTIR spectra of functionalized AuNPs and AuNS. FTIR spectra of AuNPs and AuNS functionalized with (**a**) 2 kDa PEG, (**b**) 6 kDA PEG, (**c**) chitosan and (**d**) melanin.


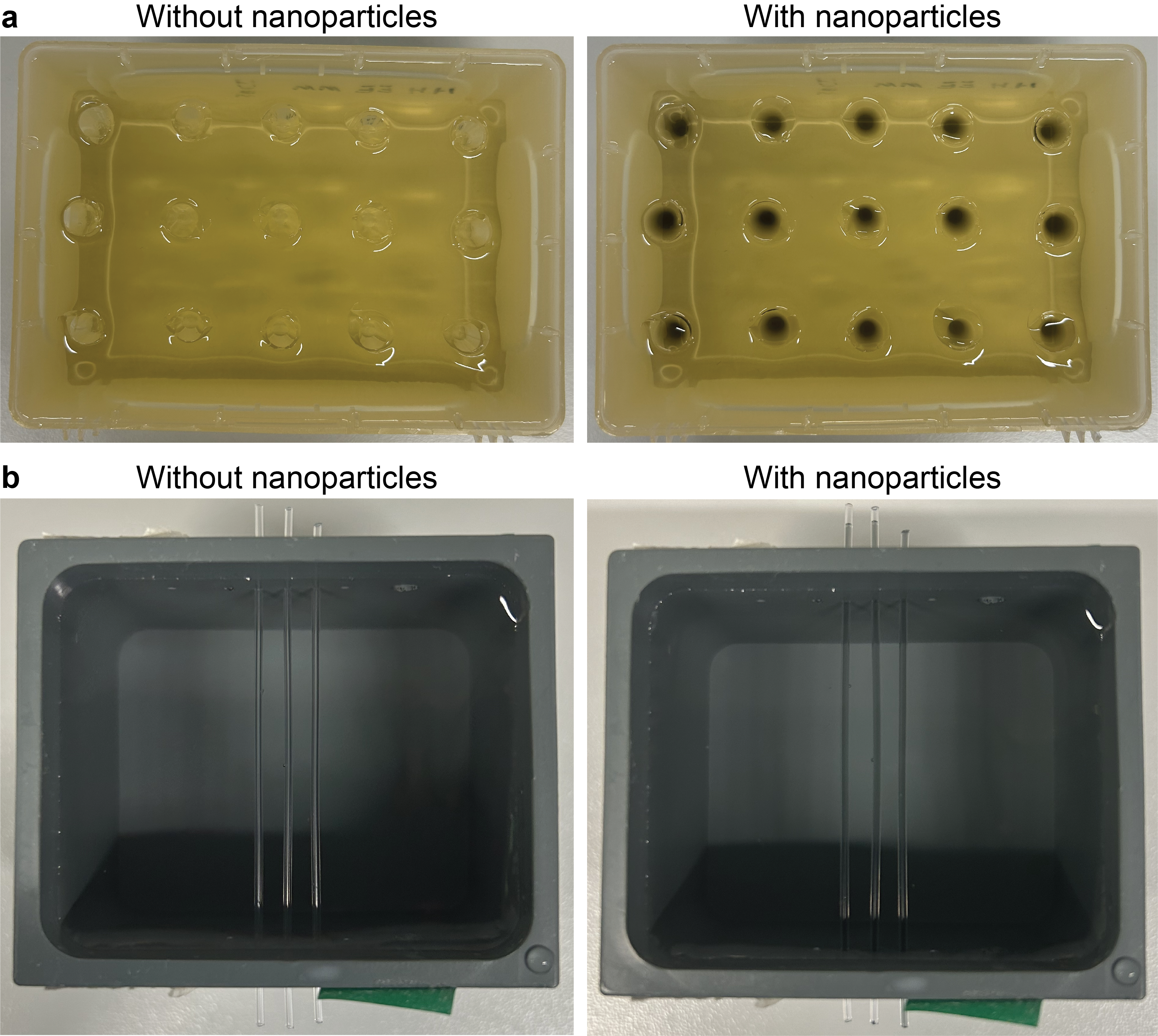


Figure S7. Phantoms used for PA imaging characterization. (**a**) Homemade gelatin phantom, and (**b**) low density polyethylene tubes used for the photoacoustic experiments before and after nanoparticle loading.


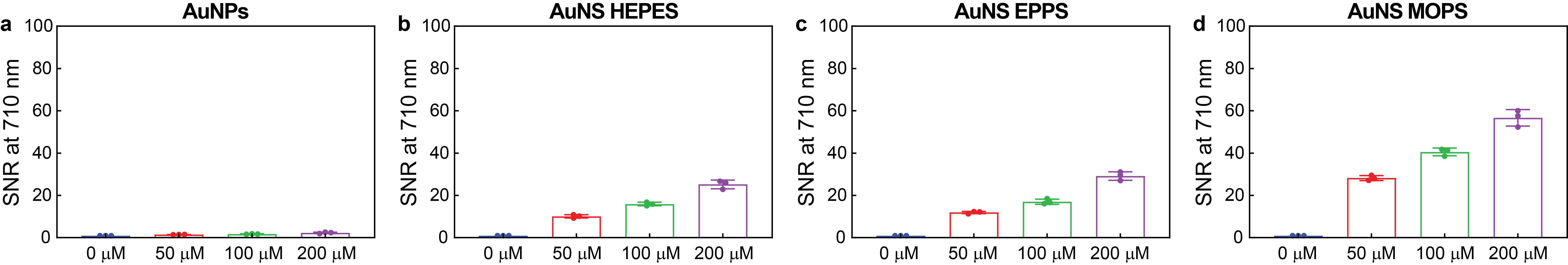


Figure S8. Signal-to-noise ratios (SNRs) of PA intensities of AuNPs and AuNS. SNRs of PA intensities at 710 nm of (**a**) AuNPs, (**b**) AuNS HEPES, (**c**) AuNS EPPS, and (**d**) AuNS MOPS at different gold concentrations (from 0 to 200 μM). Values in columns represent mean ± standard deviation of three different batches of nanoconstructs.


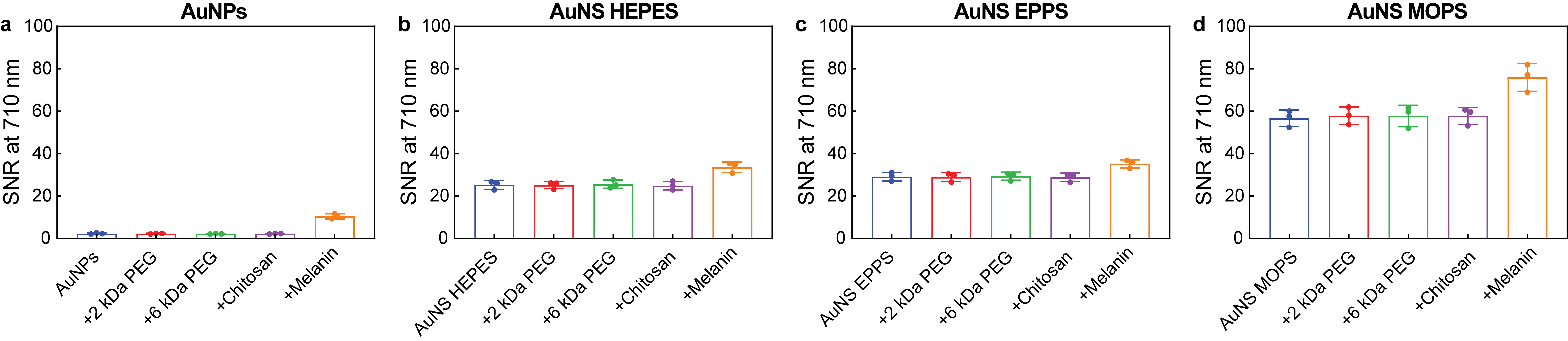


Figure S9. SNRs of PA intensities of functionalized AuNPs and AuNS. SNRs of PA intensities at 710 nm of functionalized (**a**) AuNPs, (**b**) AuNS HEPES, (**c**) AuNS EPPS, and (**d**) AuNS MOPS at gold concentrations of 200 μM. Values in columns represent mean ± standard deviation of three different batches of nanoconstructs.

| Table S7. Variations of PA intensities. Variation coefficients of PA intensities at 710 nm of (**a**) AuNPs, (**b**) AuNS HEPES, (**c**) AuNS EPPS and (**d**) AuNS MOPS at gold concentrations of 200 μM gold in polyethylene tubes. | |
| --- | --- |
|  | Variations (%) |
| AuNPs | 4.7 ± 9.3 |
| AuNPs+2 kDa PEG | 4.5 ± 4.6 |
| AuNPs+6 kDa PEG | 6.9 ± 0.4 |
| AuNPs+Chitosan | 4.7 ± 2.9 |
| AuNPs+Melanin | 1.8 ± 6.3 |
|  | Variations (%) |
| AuNS HEPES | 1.0 ± 3.2 |
| AuNS HEPES+2 kDa PEG | 3.7 ± 4.2 |
| AuNS HEPES+6 kDa PEG | 2.3 ± 3.9 |
| AuNS HEPES+Chitosan | 1.6 ± 2.0 |
| AuNS HEPES+Melanin | 2.0 ± 4.0 |
|  | Variations (%) |
| AuNS EPPS | 0.7 ± 3.1 |
| AuNS EPPS+2 kDa PEG | 5.1 ± 5.1 |
| AuNS EPPS+6 kDa PEG | 2.8 ± 6.7 |
| AuNS EPPS+Chitosan | 4.6 ± 3.8 |
| AuNS EPPS+Melanin | 2.7 ± 0.8 |
|  | Variations (%) |
| AuNS MOPS | 6.0 ± 3.2 |
| AuNS MOPS+2 kDa PEG | 3.6 ± 8.1 |
| AuNS MOPS+6 kDa PEG | 2.5 ± 2.2 |
| AuNS MOPS+Chitosan | 1.6 ± 2.8 |
| AuNS MOPS+Melanin | 0.9 ± 2.2 |


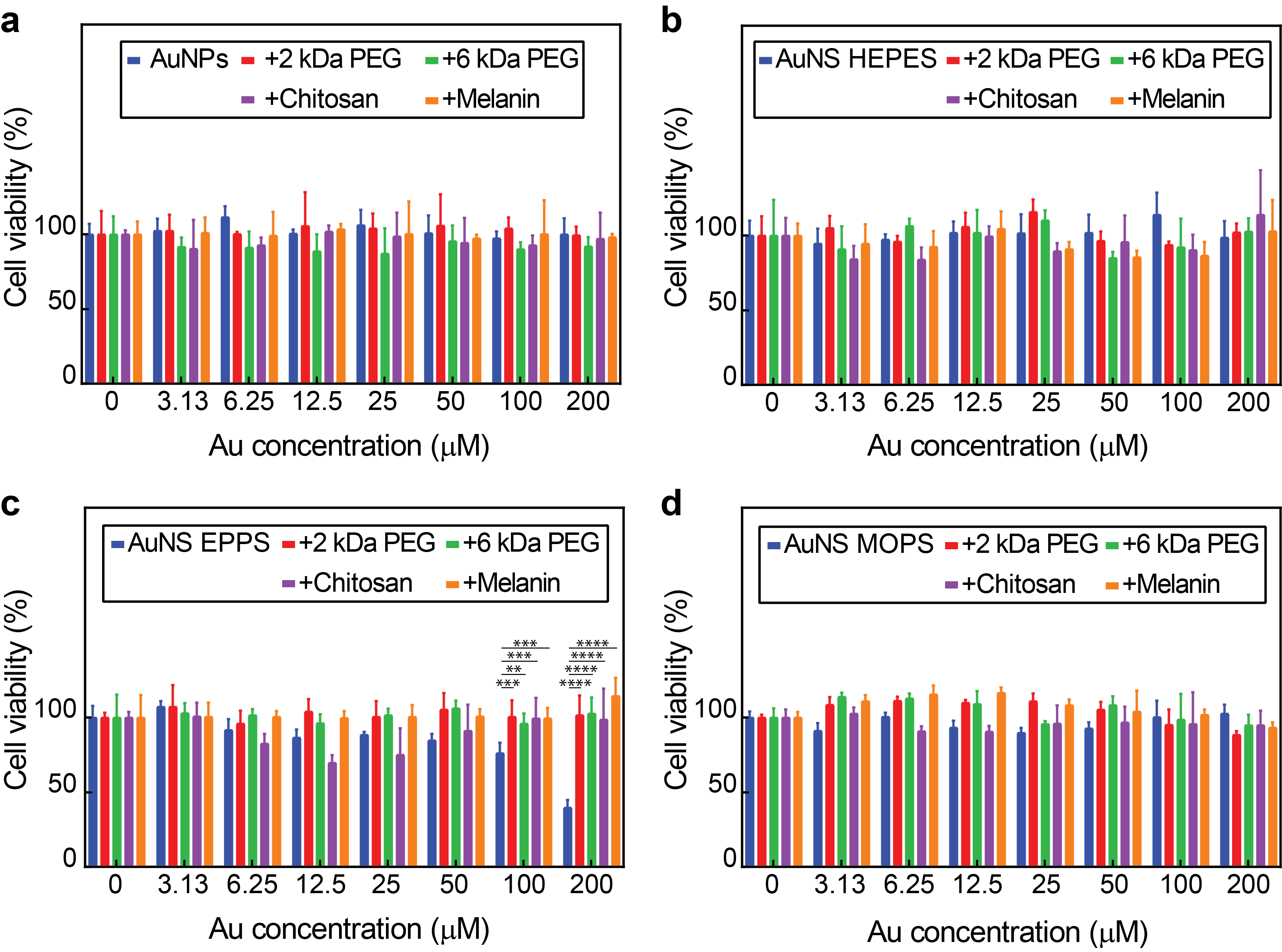


Figure S10. Cytotoxicity of AuNPs and AuNS. Cell viability of HEK 293 cells after 24 hours of incubation with (**a**) AuNPs, (**b**) AuNS HEPES, (**c**) AuNS EPPS and (**d**) AuNS MOPS (from 0 to 200 μM of gold). (**), (***), and (****) indicate groups that are significantly different with p < 0.01, p < 0.001, and p < 0.0001, respectively (two-way ANOVA).


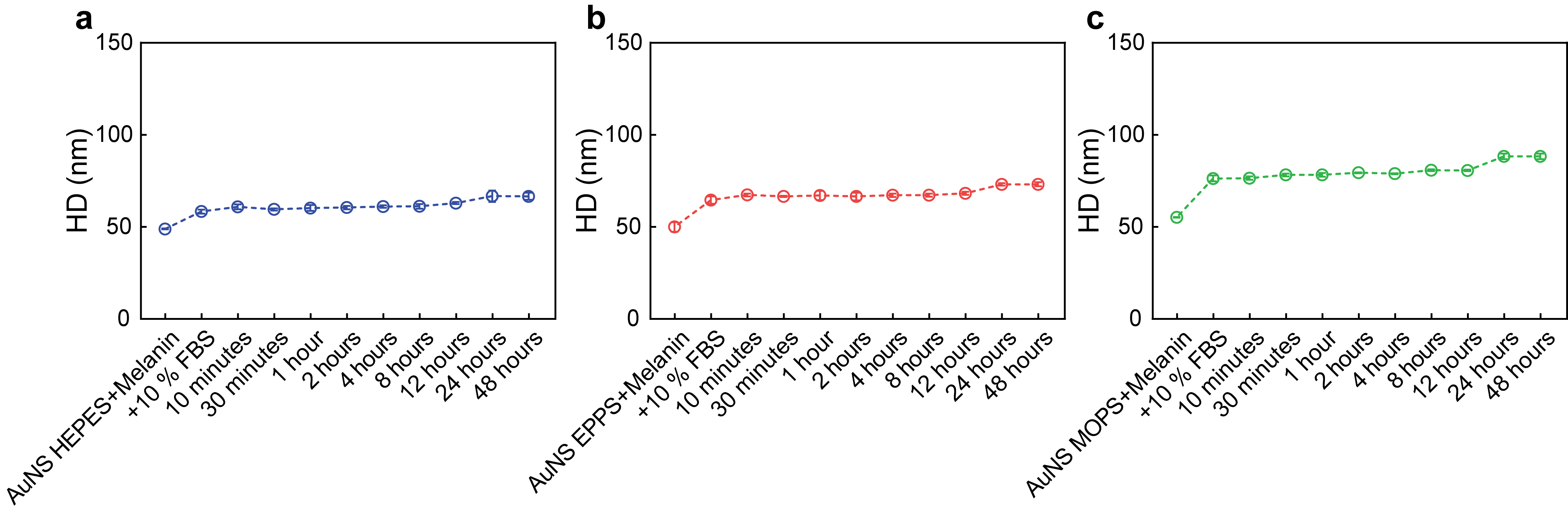


Figure S11. Stability of melanin-coated AuNS in 10 % fetal bovine serum (FBS). Hydrodynamic diameter (HD) of (**a**) melanin-coated AuNS HEPES, (**b**) EPPS and (**c**) MOPS in 10 % FBS at different incubation times.


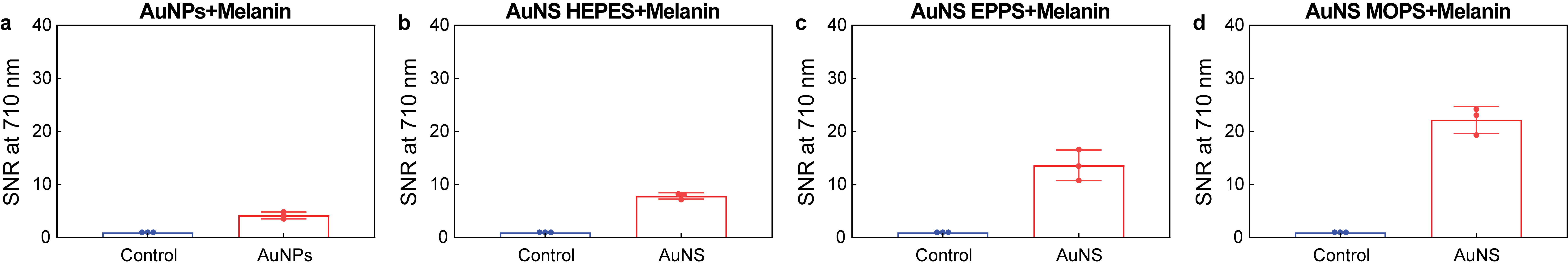


Figure S12. SNRs of PA intensities of melanin-coated AuNPs and AuNS in ex vivo imaging. SNRs of PA intensities at 710 nm of melanin-coated (**a**) AuNPs, (**b**) AuNS HEPES, (**c**) AuNS EPPS and (**d**) AuNS MOPS intramuscularly injected (50 mL, 0 or 200 μM gold) in the legs of deceased mice. Values in columns represent mean ± standard deviation of three different batches of nanoconstructs.
